# An internet-based self-applied rat phobia treatment using a Virtual Therapy Assistant: Study protocol for a randomized controlled trial

**DOI:** 10.1371/journal.pone.0281338

**Published:** 2023-02-15

**Authors:** Sonia M. González-Lozoya, Victoria Meza-Kubo, Alejandro Dominguez-Rodriguez, Cristina Ramírez-Fernández, Eduardo Bautista-Valerio, Liliana Moreyra-Jiménez, Alberto L. Morán

**Affiliations:** 1 Facultad de Ciencias, Universidad Autónoma de Baja California, Ensenada, Baja California, Mexico; 2 Área de Ciencias de la Salud, Universidad Internacional Valenciana, Valencia, Spain; 3 Tecnológico Nacional de México/I.T. Ensenada, Ensenada, Baja California, Mexico; 4 Facultad de Estudios Superiores Iztacala, Universidad Nacional Autónoma de México, Mexico, Estado de México, Mexico; Danube Private University, AUSTRIA

## Abstract

Specific phobias are a common anxiety disorder that deteriorates the lives of people who suffer from them. To reduce the symptoms produced by this mental disorder exposure therapies have been used. However, low- and middle-income countries, including Mexico, have the lowest rate of treatment due to multiple barriers that prevent addressing mental health problems. Self-applied treatments have been explored in previous studies, nevertheless, high dropout rates are a common problem in this kind of treatment. An alternative is using immersive self-applied treatments that could help increase adherence to the treatment. This article aims to present a study protocol to explore the feasibility of an Internet self-applied exposure treatment for rat phobias, using four types of immersive multimedia elements: images, videos, video games, and 360° videos. Also, the satisfaction and perception of a Virtual Therapy Assistant (VTA) that provides information and support to the user are described. The study protocol will compare two groups of participants, one on the waiting list, and the other will receive the self-applied treatment for rat phobia supported by the VTA. For this study, 45 participants will be recruited and the evaluation measures will be taken at four different times: baseline, post-treatment, and follow-ups at 3 and 6 months. The levels of anxiety and avoidance of the user manifested during the exposure to the multimedia elements, the improvement of the user’s clinical symptoms, the level of satisfaction, the perception of effectiveness, and ease of use of the self-applied system will be evaluated. This study is expected to support the viability of self-applied treatment for rat phobias and the use of a VTA, showing the impact on treatment adherence. To the best of our knowledge, this is the first study to explore an exposure treatment for rats using different multimedia elements with the support of a VTA. The work will serve as a basis for the development of new virtual assistants that help in the treatment of other types of specific phobias. This research has been registered in Clinical Trials NCT (NCT05081323).

## Introduction

The term *Specific Phobia* (SP) refers to an intense, irrational fear of something that poses little or no actual danger. Some people suffer from a phobia of specific objects or situations such as animals, heights, thunder, darkness, or closed spaces [1]. According to the World Health Organization (WHO), SP has a high prevalence in the general population, particularly animal phobia [2]. In Mexico, SP has an earlier age of onset, mainly in childhood and it persists into adulthood, women are more likely than men to develop an SP, and animal phobia is the most common along with the blood injection injury phobia [3]. People with SP tend to show a more significant deterioration in their life compared to people who have other types of phobias such as agoraphobia or social phobia [2]. Further, it has been reported that only 8.0% of people with SP seek or receive treatment [4]. Furthermore, low and middle-income countries have the lowest rate of treatment for SP (9.6%), followed by upper-middle-income countries (16%) and high-income countries (30.1%) [2]. Specifically, Mexico has a low rate of treatment for SP, and this can be observed both in the adult and adolescent populations [3].

Likewise, most mental health research is carried out in high-income countries, so there is an imbalance that must be corrected so that low and middle-income countries have cost-effective and culturally appropriate strategies to address mental health needs [5]. In addition to this, there is an imbalance between public spending on mental health and the broad treatment gap for mental disorders in Latin America [6]. To address the treatment gap for mental health problems in low- and middle-income countries, the use of Internet-based interventions has been proposed as a way to reduce cost and waiting time, and increase service availability, as these can be guided with a minimum amount of therapeutic attention, which allows a greater number of people to receive treatment and be benefited [6–8].

Additionally, psychological interventions have been implemented using exposure techniques supported with multimedia elements as an alternative to applying traditional exposure therapy (e.g. exposure to the feared stimulus, such as spiders or cockroaches) since it allows people to experience feared situations safely, maintain a high degree of control in stopping, repeating and/or prolonging the exposure time, as well as modifying and creating different actions through images and videos [9], serious games [10], and the use of immersive images or videos [11]. The exposure treatment supported by multimedia elements can be applied along with a therapist, even though there are available automated self-guided treatments that provide a complete treatment without the assistance of a therapist. In the study of Campos et al. (2019), the researchers demonstrated the effectiveness of an Internet-based exposure treatment to treat the phobia of flying, including exposure scenarios composed of real images and sounds [12]. Also, Hnoohom and Nateeraitaiwa (2017) proposed the use of a virtual reality-based smartphone application so that the user could face feared situations and animals anywhere, even in their home [13]. Furthermore, Piercey et al. (2012) investigated the effectiveness of exposure to virtual reality in treating arachnophobia using a three-dimensional video game on a desktop computer using red/blue anaglyph lenses, serving as self-help without considering the presence of a therapist or researcher [14]. The researchers successfully found fear responses and their decrease through physiological reactions (heart rate level) in the participants. However, one of the main challenges posed by self-administered interventions is reducing the dropout rate, since it is difficult to engage participants, which leads to attrition and non-completion of treatment, with a weighted average of 31 to 32% [15]. Therefore, it is necessary to consider other elements that are attractive and that motivate participants to engage with the treatment to complete it.

One option to increase a patient’s adherence to self-applied treatments could be a Virtual Therapy Assistant (VTA). The VTA is an application focused on the area of health therapies that uses interaction with the user based on voice commands. The use of VTA’s has expanded considerably in recent years, due to the high availability of smart devices that facilitate various daily tasks, require little cognitive learning load, and are accessible to many people [16, 17]. Some studies have shown that the use of VTAs applied to health sector technologies brings benefits such as adherence to oral anti-diabetic drugs in older people [17], or helping patients with dementia and memory loss to identify and remember critical memories [18], and to practice cognitive reconstruction exercises to decrease public speaking anxiety [19].

The phobia of rats (musophobia) is among the top five most common animal phobias [20, 21], and rats are considered to be the most important invasive species worldwide [22]. However, to the best of our knowledge, no multimedia-based exposure treatments have been proposed, reflecting the need to develop treatments aimed at this type of phobia.

This article aims to present the protocol to evaluate an Internet-based Self-applied Treatment for Rat Phobia with the support of a VTA, using four types of multimedia elements for each level of immersion (images, videos, video games, and 360° videos). For more details about the proposal, see [23]. The protocol describes the plan to explore the effectiveness of the treatment, as well as the satisfaction and perception of the effectiveness and ease of use of the VTA. Therefore, it is proposed to verify if the participants show a reduction in the self-reported symptoms of fear, anxiety, and avoidance in the presence of the rats individually, when comparing before and after the intervention with the self-applied treatment supported by the VTA; as well as reporting measures of satisfaction and perception of effectiveness and ease of use of the participant towards the VTA.

## Methods and analysis

### Study design

**Fig 1:**
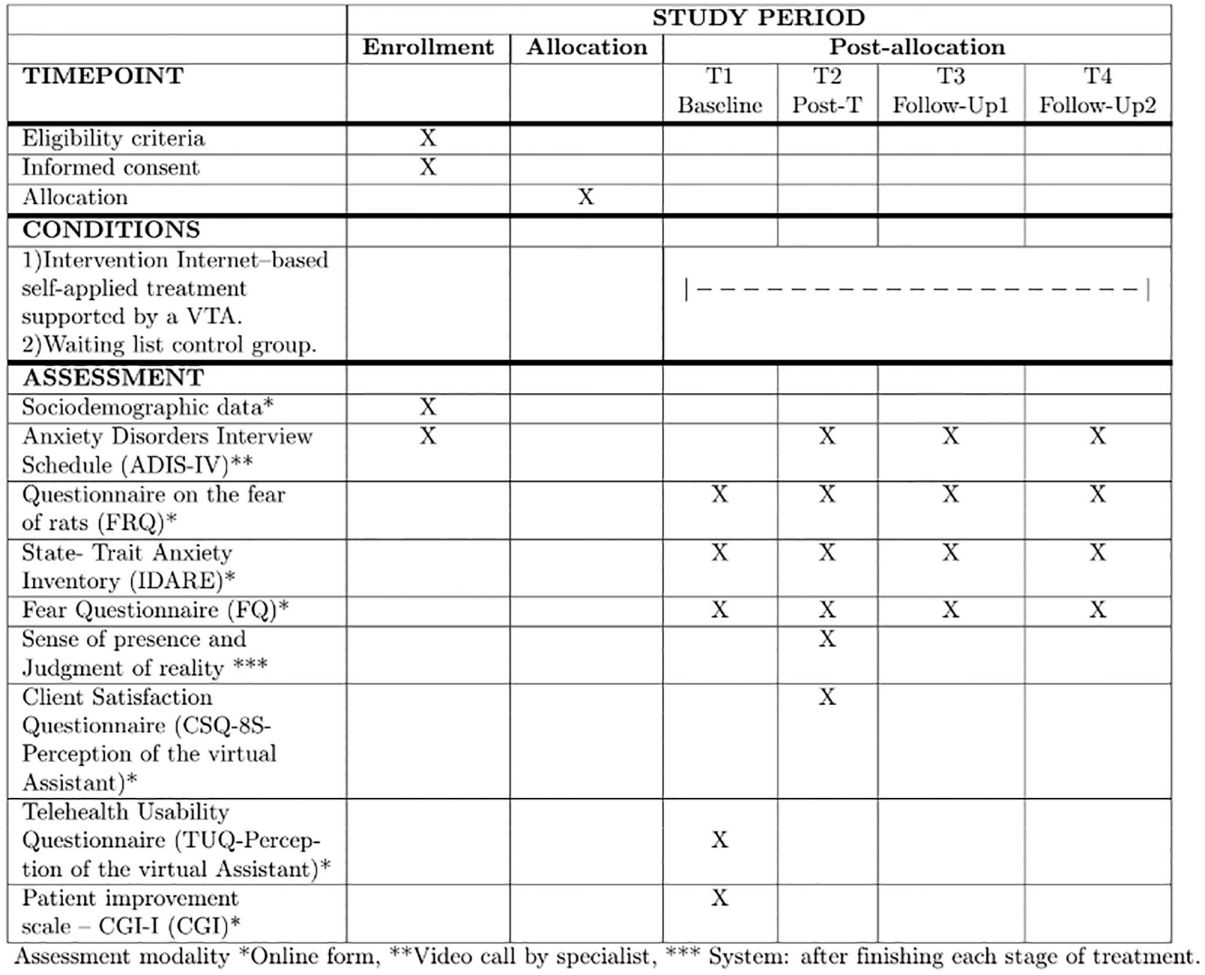
SPIRIT schedule presenting participant timeline.

A randomized controlled clinical trial including two groups, experimental and control groups will be implemented. The study will include inter-subject measurements. For the experimental group, four evaluations will be performed: baseline, post-treatment, and follow-up at three and six months. Fig 1 shows in detail the instruments that will be applied and the times of their application.

The control group will carry out five measurements, pre-time period waiting list, post-time period waiting list, post-treatment, follow-up at three months, and follow-up at six months. Participants will be randomly assigned to one of the two groups:

1. Internet-based Self-applied Treatment supported by a VTA, where the participants will be gradually exposed to virtual rats through four types of multimedia elements (images, video, video game, and 360° videos) defined by four stages, with the support of a VTA that will provide information and help the phobic patients during all treatment.
2. Waiting list control group, where participants will receive the treatment after 1 month.

**Fig 2:**
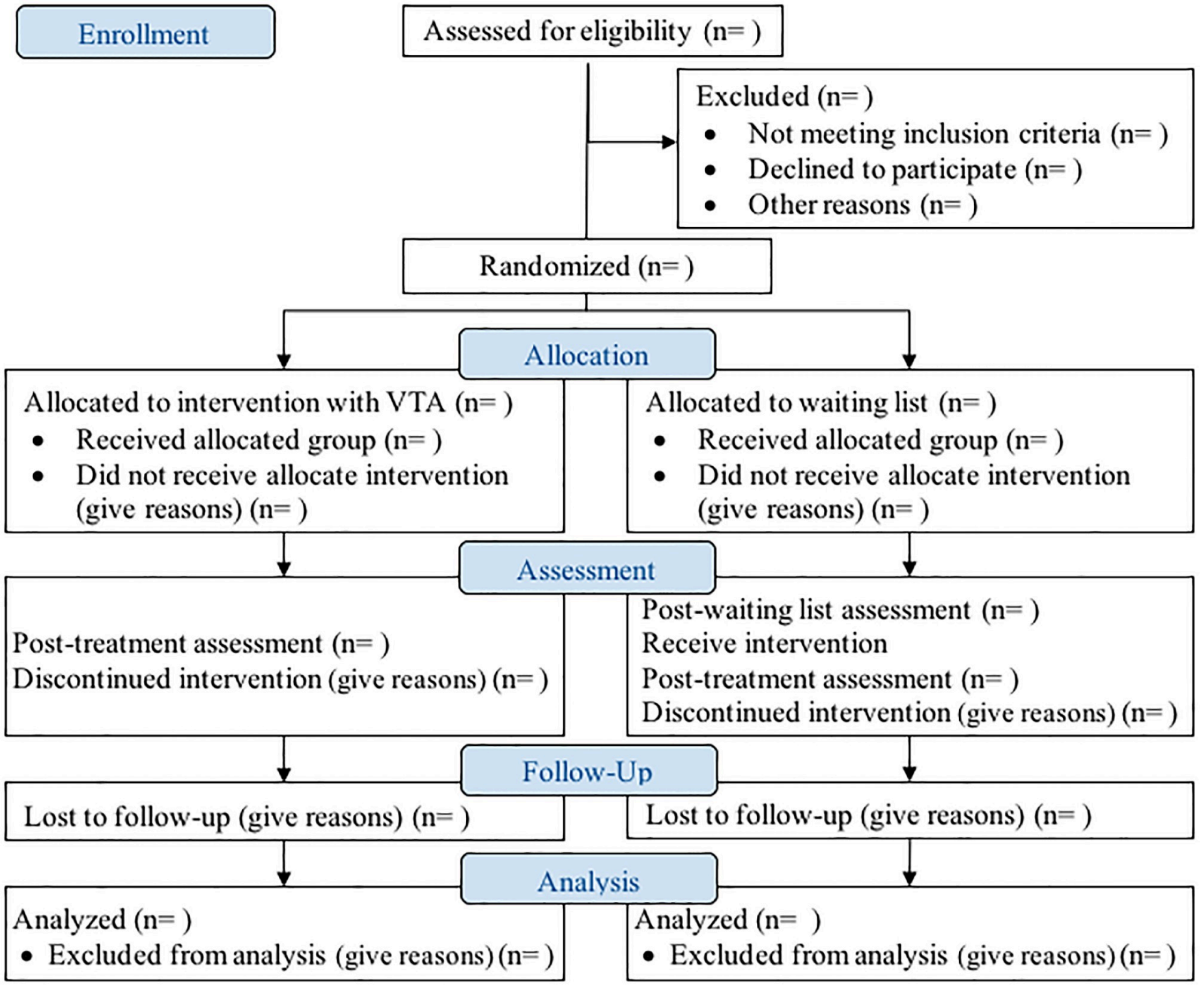
Study design flow. Flow chart of the study design of the self-applied treatment supported by the Virtual Therapy Assistant based on the Consort 2010 Flow diagram.

Post-treatment and follow-up will be applied to all the participants in both groups to analyze the efficacy of the intervention (see Fig 2). The protocol for this trial and supporting CONSORT checklist are available as supporting information, see the document S1 Checklist and protocol.

### Randomization process

The participants will be assigned to one of the two groups using permuted blocks obtained through the software Study Randomizer [24]. The process will consist of a researcher of the team creating the permuted blocks previously, and as soon as a participant complies with all the inclusion criteria points and none of the exclusion criteria points, he/she will be assigned to the corresponding condition.

### Sample size calculation

Sample size calculations are based on the number of participants and the expected power size considering previous Virtual Reality, self-applied, Internet-based interventions presenting virtual objects, such as spiders, to patients with arachnophobia [25]. The total sample size and the statistical power were calculated using the G*Power software [26] for repeated measures between factors: MANOVA. A priori power analysis was performed. We assumed a medium effect size (0.25). Considering a significance level of *α* = 0.05 (two-sided) and a statistical power of 1 − *β* = 0.80, a sample of *n* = 38 participants is estimated (Actual *power* = 0.84). Also, 7 participants will be added to this estimated sample size to account for an assumed 36% dropout rate, considering [27] mean meta-analytic attrition rate. Therefore, the total number of participants in this study will be *N* = 45, similar to [25], and equal to [28], which includes an online intervention with two groups.

### Participants, recruitment, and eligibility criteria participants

The participants will be 45 adults according to Mexican law who volunteer to participate in it. The study will be published in different media such as social networks (Facebook, WhatsApp, and Twitter) and other media of the institutions of the participating researchers for dissemination and to reach more potential participants. Potential participants may contact the study recruiters by email, WhatsApp, or phone call. Any Mexican person who understands Spanish can benefit from the intervention, as long as they meet the following inclusion and exclusion criteria.

Inclusion criteria:

- Have a mild or moderate degree of phobic intensity according to the diagnostic criteria for a specific phobia of rats.
- Have essential digital knowledge and skills (computer and Internet use).
- Have the basic technologies and technological devices: email, computer/laptop, smartphone, microphone, Internet connection, and Bluetooth.
- Sign the informed consent.

Exclusion criteria:

- Receive additional psychological or psychopharmacological treatment.
- Being diagnosed with an additional type of anxiety disorder or psychopathology.
- Present any medical condition that may put the participant’s life at risk (heart disease, respiratory disease, pregnancy, among others).

The participants must meet all 4 points of the inclusion criteria and do not have to meet any of the 3 points of the exclusion criteria to receive the intervention hardware.

### Blinding and unblinding

The participants will not know that another group or condition exists, knowing only the agreed intervention date. While the evaluation staff cannot be blinded to the assigned condition of the participants, they are therefore advised not to reveal such information. In the case where blindness cannot be maintained with a participant, either because he or she has communication with another participant or another reason that reveals his or her condition, the condition that most benefits him or her will be assigned and the ethics committee will be notified in this regard and will also be reported in the results.

### Ethical considerations

This study will follow the ethical guidelines of the Declaration of Helsinki (WMA; World Medical Association). Also, this research has been approved by the Ethics Committee of the Autonomous University of Baja California (Mexico) (POSG / 021–1-03) and has been registered in Clinical Trials NCT (NCT05081323), who will be notified of any changes in the protocol. The security and confidentiality of the participant’s data will be maintained by assigning an identification sheet, and only the researchers responsible for the study will have access to the information. All participants must accept written informed consent before answering the initial assessment and being randomly allocated to one of the two groups.

Participants will be assessed individually and only staff trained in the application of instruments will have direct contact with the participant. They will be informed before proceeding with any application about the use of the data, the freedom they have to abandon the study if they wish, and finally, they will decide whether or not they wish to participate in the protocol giving their authorization or rejection in writing to the evaluator. A personal file will be made of those who participate in the application of the tests. All files will be stored according to ethical conditions with full support and only the principal investigator will have access to confidential data.

Once the study data has been collected and the data analysis has been carried out, the results obtained will be published in journal articles, and also these will be reported within the Clinical Trials NCT (NCT05081323). Data will be available from the lead author upon request.

Substantive contributions to the design, conduct, interpretation, and reporting of a clinical trial are recognized through the granting of authorship on the final trial report.

### Materials

To carry out the study, we designed the scenario and the first prototype of the VTA for a gradual exposure treatment for rat phobias of medium fidelity, which allows:

- Oral and/or written verbal interaction through a chat using voice recognition and synthesizer.
- The visualization of phobic stimuli through images, videos, video games, and immersive 360° videos.
- Real-time heart rate and avoidance behaviors monitoring.
- Self-assessments of the participant on their perception of fear and avoidance caused by the exposed phobic stimulus, that is, obtaining subjective units of fear and avoidance.
- Execution of actions requested by the participant or established according to his/her reactions to provide a personalized treatment advance.

For the prototype to become accessible, replicable, and self-applicable, we consider the use of easy-to-acquire, low-cost, and popular devices to reduce the cognitive load. For this, it is necessary to include:

- A mobile or desktop PC with a webcam and microphone for verbal interaction, exposure to phobic stimuli, self-assessments, and frontal video capture of the patient.
- A Mi Smartband to obtain the heart rate of the participant.
- A keyboard or joystick for mobility and virtual interaction with the video game in the third stage.
- A smartphone and cardboard to watch the immersive videos in the fourth stage.

### Intervention

The intervention consists of four stages that will use different visual elements to represent and replace a rat in a gradual exposure treatment for SP to this animal. As the order of the stages increases, the intensity of the phobic stimulus increases so that during each stage, the participant is gradually exposed to a certain number of elements representing a phobic situation or object. In this sense, gradualness considers an approach with realism, interaction, and intensity [29].

During the first and second stages, the participant will be exposed to the phobic object supported with multimedia material (visual and audiovisual) based on the Multimedia Behavioral Avoidance Test (MBAT) instrument. The MBAT is a reactive test where the participant is presented with various visual stimuli (images and videos) related to the object of fear [30]. The purpose of the first two stages is that the user could get used to and tolerate the appearance of rats by observing ten images of approximately 10 seconds each and seven short videos with an approximate 1-minute duration. This procedure is adapted from Ruiz-García and Valero-Aguayo (2020a) [9].

While in the third stage, the participant will be exposed to a video game consisting of five levels. Each level increases the intensity of exposure through modifications of the appearance and size of the virtual rat. The video game’s plot takes place in a house where the user must explore, find and catch rats to go to the next level. The objective is that the participant can approach, observe, and interact with different representations of virtual rats during the game. The duration depends on overcoming the levels of the video game for each participant, however, the session cannot last more than an hour. Some related works have shown that games are effective in treating other types of specific phobias, such as dog or spider phobias [13, 31].

In the fourth stage, the participant will be immersed in a virtual reality environment simulating a natural situation (e.g., Feel Good COVID [32] through 360° videos, using a smartphone and Google cardboard). The video content will show rats exhibiting different types of natural behavior. The purpose of this stage is that the user can immerse himself or herself and keep watching the street rats around the scene.

In the middle and at the end of each stage, the participant will evaluate himself/herself by answering the following: According to your perception and with the greatest sincerity, using a scale from 1 to 10, where 1 is nothing and 10 is a lot, 1) “Degree/intensity of avoidance that you consider you experienced during the exposure” and 2) “Degree/intensity of anxiety that you consider you experienced during the exposure”.

The intervention will also have a VTA that will guide and inform through monitoring the participant’s reactions. In addition, the VTA will propose deep breathing exercises to reduce any inferred alteration, at the following times: a) at the beginning of each session, b) when it detects any alteration in the heart rhythm, or c) if it is requested by the participant. During the exercise, the VTA will provide support with instructions and images of the steps to be carried out, and by monitoring the heart rate, it will also verify if the participant is fit to continue with the therapy. To do this, in Table 1, heart rate monitoring is classified into four states considering heart rate zones: Normal, the minimum value varies from 40 or 60 beats per minute (bpm), depending on the physical condition of the participant, to threshold A; Mildly altered, greater than threshold A to threshold B; Highly altered, greater than threshold B to threshold C; and In a crisis, greater than threshold C [33, 34]. Thresholds vary based on 50%, 65%, and 85% of each participant’s maximum heart rate (see Table 2).

**Table 1:** Classification of the HR status.

Stage	Rate of bpm
Normal	PC: between 40 bpm to A NPC: between 60 bpm to A
Mildly altered	Greater than A to B
Highly altered	Greater than B to C
Crisis	Greater than C

PC: physical condition, NFC: no physical condition

**Table 2:** Variables considered in the HR classification.

Variables	Value
Age	Participant age
MHR	MHR = 220—age
Threshold A	A = (MHR * 0.50) bpm
Threshold B	A = (MHR * 0.65) bpm
Threshold C	B = (MHR * 0.85) bpm

MHR: Maximum Heart Rate.

Considering that the participants are exposed to phobic stimuli, it is determined that the mildly altered state is normal in the phobic participants for the treatment to be effective; if it detects a highly altered state, then the VTA will pause the exposure to the phobic stimulus and will recommend a relaxation strategy (in this case deep breathing) until the pulsations normalize; if the state is in crisis, or a slower than normal heart rate is detected, then the VTA will recommend completely stopping the exposure to the phobic stimulus until the participant is in a normal state, and the continuity of the session will depend on the participant’s decision.

#### Setting

**Fig 3:**
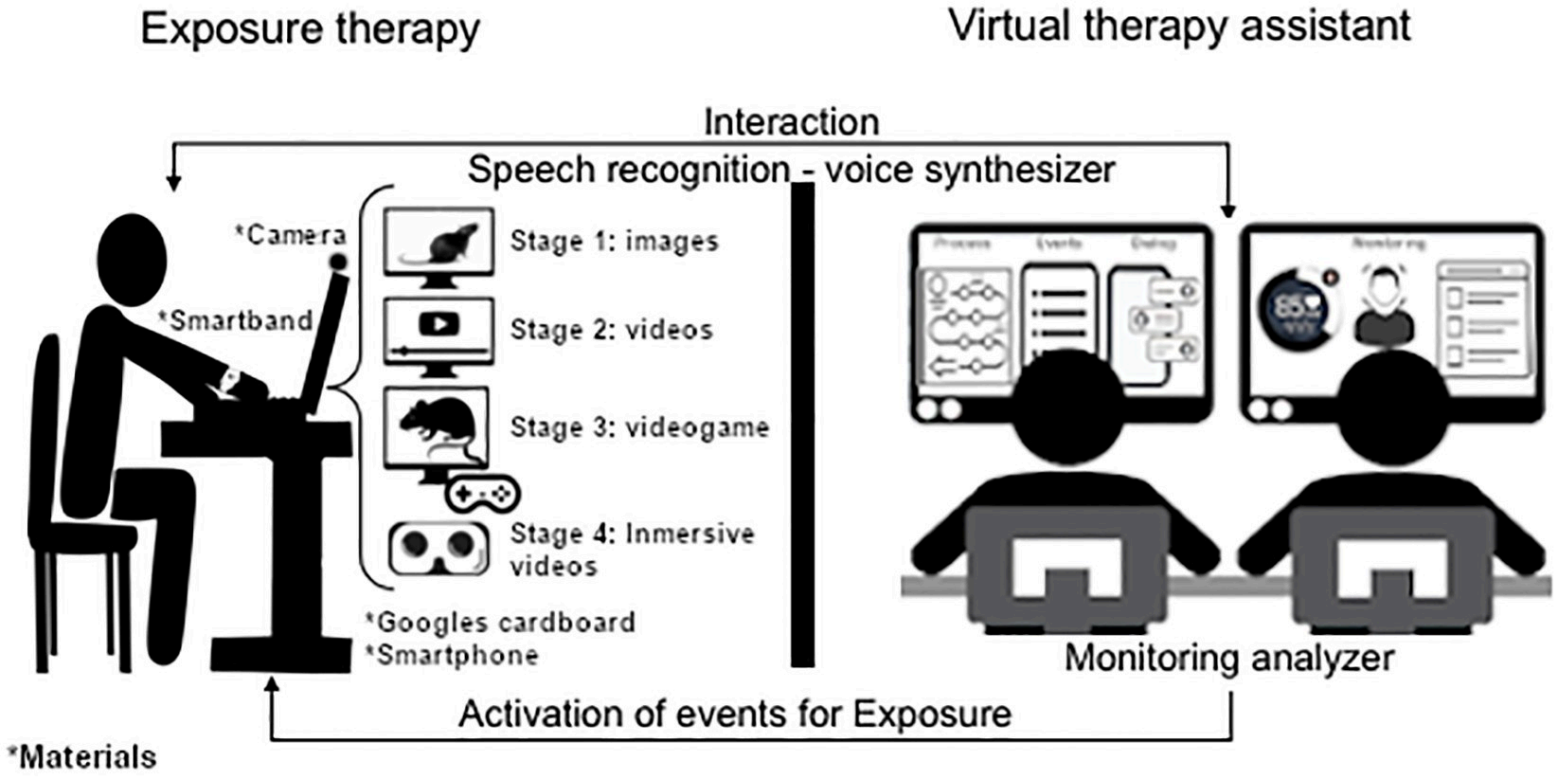
Intervention setup. Semi-controlled intervention scenario in a laboratory using the Wizard of Oz technique in case of a failure or unconsidered events.

The intervention will be carried out in a semi-controlled way in a laboratory where the participants will present themselves. In each session, there will be the support of an on-site therapist to ensure the well-being of the phobic participants (see Fig 3). The setting is described below:

The participants will be alone in the laboratory, wearing a smart band to monitor their heart rate throughout the intervention, and will sit comfortably in front of the screen (with Camera) where they will be shown the visual phobic stimuli of the treatment. During the intervention, the camera will identify avoidance behaviors with the help of face and eye detection algorithms. In addition, they will have at hand a smartphone with immersive glasses to watch the 360° videos for the fourth stage.

An evaluation technician will be located next to the laboratory, ensuring the collection of data and the correct operation of the VTA. The technician will maintain a communication channel through dialogues using a voice chat that will use voice recognition to detect the commands requested by the participant, a voice synthesizer to transmit the VTA messages, and will determine the progress of the treatment according to the data obtained through the follow-up. The structure of the VTA dialogues is as follows: content and objective of each exposure therapy, description of each element to be exposed, preparation of the participant before the exposure, a proposal for implementation, and step-by-step instructions for the deep breathing exercise. Also, recognition of keywords such as next, previous, remove/eliminate, and in the case of the video game and video stages: finish, pause, and continue. To prevent failures or unconsidered events, the technician with the support of the therapist can handle the situation using the Wizard of Oz technique [35], and these situations can be considered as feedback in the dialogue and/or events of the VTA. The duration of the intervention varies according to the progress of the treatment for each participant, setting a limit of 4 weekly sessions. As a retention plan for the participant, apart from providing personalized treatment and psychological attention if needed, they will also be offered scheduling, financial support for transportation to the place of intervention, soft drinks, and snacks. Participants may withdraw from the study for any reason at any time. The investigator also may withdraw participants from the study to protect their safety. In case of desertion, they will be asked the reasons for their decision.

### Psychological measures

#### Diagnostic evaluation data

- Sociodemographic data: collect and record contact data, sociodemographic information (age, gender, marital status, school level, and current employment situation), medical history (to rule out risk diseases and psychological / psychopharmacological treatments), availability and basic knowledge about technological devices (computer, smartphone, and tablet, among others).
- Anxiety Disorders Interview Schedule (ADIS-IV) [36]: it is a semi-structured interview based on the DSM IV diagnostic criteria. It evaluates the degree of fear and avoidance of certain situations in the specific phobia. This allows for establishing a differential diagnosis between certain anxiety disorders, mood disorders, substance abuse, and somatoform disorders. ADIS-IV will be performed by a trained psychologist who will determine the severity of the patient’s condition on a scale of 0–8, where 0: no phobia, 1–3: mild phobia, 4–5: moderate phobia, and 6–8: severe phobia.

#### Pre / post and follow-up (3 and 6 months)

- Questionnaire on the fear of rats (FRQ): it is a questionnaire that has been adapted from the Fear of Spider Questionnaire (FSQ [37]), for this, the word “spiders” has been modified to “rats”, and the questions were modified to evaluate the phobia of rats [38]. The FSQ has been adapted to Spanish by Forcadell et al. (2018) and has shown adequate reliability (Cronbach’s alpha of 0.966) [39].
- State-Trait Anxiety Inventory (IDARE) [40]: The version in Spanish of the STAI (State-Trait Anxiety Inventory) consists of 40 statements; 20 evaluate trait anxiety and 20, state anxiety. It has been validated in Mexico by Rojas-Carrasco (2010), obtaining adequate reliability for the two anxiety dimensions (Cronbach’s alpha coefficients greater than 0.83) [41].
- Fear Questionnaire (FQ) [42]: it assesses the intensity with which certain situations are avoided and concerned, and it also contains a scale where the patient rates the present state of their phobic symptoms.

#### Post-treatment

- Sense of presence and judgment of reality: this instrument evaluates the degree of presence experienced through the visual elements of a 360° video, using two questions with a Likert scale from 0 to 10 (0 = nothing, 10 = too much) [43]: “To what extent have you felt present in the immersion?”, “To what extent have you felt in a place in which the rats have appeared?”.
- Client Satisfaction Questionnaire (CSQ-8S): it consists of 8 questions with a total score ranging from 8 to 32 to measure the level of satisfaction with the treatment and has been used to evaluate self-applied treatments [8]. The question “To what extent did you find it aversive?” was added to this questionnaire [44].
- Telehealth Usability Questionnaire (TUQ): it assesses the usability of telehealth systems [45], through two dimensions: 1) effectiveness, and 2) ease of use. It contains 12 questions with a 7-point Likert scale (1 = *disagree*, 7 = *agree*). The questionnaire was translated into Spanish with reliability values of 0.9060 in effectiveness and 0.94409 in ease of use [46].
- Patient improvement scale- CGI-I (Global Clinical Impression Scale, CGI) [47]: it contains an item that was adapted from the CGI scale and that allows the patient to assess his/her degree of improvement on a 7-point Likert scale (1 = much better, 7 = much worse).

### Study period

The initial evaluation of the participants is planned to start in January 2023 and we expect to conclude in June 2023. For this date, we expect that 45 participants that fulfill the criteria will be included. Also, they will be included in 3-month and 6-month follow-up assessments to evaluate whether the intervention results are maintained over time.

### Proposed statistical analysis

To confirm or reject the established hypotheses, t-student tests will be performed for paired samples, comparing the averages of each symptom before and after the intervention of the proposed treatment. And with this, to be able to accept or reject the null hypotheses establishing that “The self-applied treatment supported by the VTA does not reduce fear/anxiety/avoidance of the participant in the presence of the rat’”. Likewise, to compare the effectiveness it will be examined whether the intervention reduces the targeted symptomatology (fear, anxiety, and avoidance) in the presence of the rat, performing Multivariate analysis of Variance (MANOVAs), along with partial eta squared to calculate the effect size measure [28, 48] will be conducted (within-group comparisons; Time 1 [T1]—Baseline, Time 2 [T2]—Post-treatment, Time 3 [T3]—3-month follow-up, Time 4 [T4]—6-month follow-up). Also, post hoc tests (Tukey HSD) [49] will be conducted, along with between-group comparisons with experimental and control groups carried out from Time 1 to Time 4. Additionally, a descriptive statistical analysis will be made to evaluate the satisfaction and the perception of effectiveness and ease of use of the VTA. The participants will be requested to fill out all the questionnaires to initiate the intervention and to reduce or avoid missing data. Also, to comply with the same points at post-test and follow-ups, a therapist will evaluate the completeness of the instruments right after they were applied. The missing data or participants that abandoned the treatment or not completed it will be analyzed using Intention to Treat Analysis [50, 51]. The analysis will be conducted with the Statistical Package for Social Sciences (SPSS) version 21.

### Possible negative effects and strategies to reduce the risk or damage for the participants

To ensure the well-being of the participants, a preliminary evaluation of the treatment was carried out with psychologists with experience in patients with phobias in the Mexican population. This evaluation consisted of receiving feedback from phobic users on the treatment proposal and obtaining their perception of usefulness and experience of use regarding each gradual exposure therapy. The intention is to strengthen the content of the treatment using different types of multimedia to represent the object of fear and improve the interaction of the VTA. The details of the evaluation process and results can be consulted in González-Lozoya et al. (2021) [23]. A possible negative effect of our proposal exists when a wrong diagnosis is made regarding the severity level of the patient’s phobia. Should this occur, it would be possible for a patient to start treatment at a stage with a high intensity of phobic exposure, where she/he could experience a state of crisis and aggravate the phobia. To prevent this type of situation, the participant is asked to fill out a form with truthful data to evaluate a diagnosis (section IV of the ADIS-IV interview for specific phobias) to obtain the level of severity of the phobia and ensure that the initial stage of exposure is correct. To ensure this, the participant will receive detailed instructions through an explanatory and instructive video that will inform the importance of capturing the user’s data as accurately as possible, along with an initial call by the therapist as reinforcement to solve doubts and receive comments about the study. In case of an adverse event during the intervention, the participant will be withdrawn from the study and will receive personalized attention from the specialist to stabilize him/her.

## Discussion

This article presents the protocol to evaluate the feasibility of a treatment proposal for participants with a specific phobia to rats, using a self-applied system guided by a VTA, along with immersive visual and auditory elements such as images, videos, a video game, and 360° videos. The study will be conducted through two conditions, a group that receives the treatment compared to a control group that is assigned to the waiting list that after the waiting period is completed will also receive the treatment. Likewise, this study seeks to explore the effectiveness of the VTA, by evaluating the level of satisfaction, and perception of effectiveness and ease of use of the self-applied treatment.

Among the strengths of this study, to the best of our knowledge, this is the first Internet-based self-applied treatment for rat phobias that integrates a VTA that would guide the treatment with patients with low to middle phobia symptomatology, and that integrates various elements of multimedia exposure. Most of the research in the literature reports treatments for SP that use fixed elements of exposure such as images, 360° images, videos, and audios [7, 11, 30], while only Donker et al., (2019) report having used 360° videos as a treatment for acrophobia [52]. This allows for more significant interaction, immersion, and realism with the virtual environment and greater control over progressive exposure to the phobic stimulus. Furthermore, a reduced number of studies have applied technology-based solutions for Exposure Therapy focused on treating rat phobias. Most of the available treatments are directed towards other types of animals and insects [9, 53] and specific situations such as fear of flying [11]. In addition to this, only a few studies integrate physiological markers along with cognitive and behavioral measurements [54, 55]. In this study, heart rate monitoring has been integrated as a physiological indicator, and face and eyes detection to determine the level of avoidance as a behavioral indicator, data that serves to monitor the emotional state of the participant, which will allow greater control and security for the user in the face of gradual exposure.

An additional strength of this study is the treatment modality, that is, a self-applied treatment based on the Internet, which may have a greater scope in the coverage of care for people with this type of specific phobias. This turns out to be relevant for the Mexican population due to the low treatment rates [3] and the limited access to mental health services in Latin America [6]. Also, this study could be helpful for other developing or low-income countries where it has been observed that 79–93% of the people with depression and 85–95% of the people with anxiety were not covered to receive treatment [56]. Also, this treatment proposal has been validated by a group of experts, and the tool is designed and developed by a team of engineers and clinical psychologists, therefore ensuring that the tool is efficient and effective in terms of usability, presence, enjoyment, and supported by theoretical concepts [23].

Regarding the limitations of the study, the main one is that only participants with low to moderate symptoms can safely receive the intervention. This limitation is related to the requirements that people with higher symptoms of animal phobia have whether they enter a critical state, such as support and supervision. Future studies should focus on the development of psychological treatments based on online interventions for people with higher symptoms of phobias.

The second limitation is related to the absence of direct contact with a therapist, which could affect the adherence and acceptability of the intervention. Therefore, the completion of the treatment would help the participants who have an animal phobia. It is expected that the VTA would help reduce this lack of adherence and help to overcome this issue by maintaining communication with the patient and providing voice instructions to help during the treatment.

A third limitation of our proposal relates to the data collection process, in which participants need to provide their symptomatology through self-report. It has been observed that self-reports lack reliability and objective data [57]. To solve this limitation, other sources of information are being integrated into this development, including physiological indicators, such as the heart rate, that could be compared to the psychological responses (e.g. fear of the animal), and analyze the reliability of both indicators.

A fourth limitation is that this protocol does not separately measure the effect of the elements of the proposed treatment. This situation implies that there will be no separated groups (e.g., a group with images, videos, video games, and immersive 360° videos). Although it would be relevant to analyze the therapeutic package elements individually to explore whether certain elements are necessary or not for the treatment, it would considerably increase the sample size and, therefore, would exceed the budget available for this study. It should be noted that this study will be carried out in Mexico, where online interventions are still scarce, and the available budget for these interventions is not as extensive as in other countries.

## Conclusion

This study explores the use of a VTA that uses different types of multimedia to provide self-applied exposure to rat phobias. This work can inform the development of a new VTA to provide exposure treatments to users with other types of specific phobias. Likewise, this study will explore the proposed treatment’s efficacy to carry out a gradual exposure treatment and evidence the use of self-applied treatments for the SP treatment. The use of a VTA that considers the user’s context to guide the progress of the exposure could help improve adherence to the treatment and thus reduce the dropout rate.

## Supporting information

S1 Checklist Consort 2010 checklist. (PDF)

S1 Protocol Study protocol. (PDF)

S2 Protocol (PDF)

S1 Appendix Informed consent form. (PDF)
