# Supplementary material for: An internet-based self-applied rat phobia treatment using a Virtual Therapy Assistant: Study protocol for a randomized controlled trial
Source: PLoS One. 2023 Feb 15;18(2):e0281338. doi: 10.1371/journal.pone.0281338 (PMC9931099; doi:10.1371/journal.pone.0281338)
Supplement: S1 Protocol — (PDF) [file pone.0281338.s002.pdf]

No. de registro 001/

1. Título del Proyecto de Intervención

Tratamiento de exposición autoaplicada para fobias a las ratas guiado por un Asistente Terapeuta Virtual

2. Investigadores/Identificación

INVESTIGADORES	No. Emp / Matrícula	Teléfono (ext.)	Correo-E
María Victoria Meza Kubo	15439	6461173317	mmeza@uabc.edu.mx
Alberto Leopoldo Morán y Solares	13399		alberto.moran@uabc.edu.mx
Cristina Ramírez Hernández			cramirez@ite.edu.mx
Alejandro Domínguez Rodríguez			alejandro.dominguez.r@campusviu.es
Sonia Maribel González Lozoya	00364101	2221170678	sonia.gonzalez68@uabc.edu.mx

3. Instituciones participantes

Institución (Razón social y dirección)	Sujetos de estudio	¿Otorgó aprobación al proyecto?
Instituto Tecnológico de Ensenada<br><br>Universidad Internacional de Valencia España	Adultos de 18-60 años de edad con algún nivel de fobia a animales pequeños. Que cuenten con una medida de severidad de fobias a las ratas entre 1 a 3 de una escala de 0 al 4, donde 0 es no fóbico, y siendo 4 el grado nivel alto de fobia.	Si<br><br>Si

4. Fuentes de financiamiento

Institucional	X	Especificar: Universidad Autónoma de Baja California
Gubernamental	X	Especificar: CONACYT, Beca a estudiante de doctorado
Comercial		Especificar:
Otro		Especificar:

5. Resumen (Límite 300 palabras)

El trabajo de investigación propone tratamiento de exposición mediante un asistente terapéutico virtual llamado VTA (siglas en inglés: Virtual Therapy Assistant), consciente del contexto que interactúa de forma verbal con el paciente, para guiar y controlar distintos tipos de terapias de exposición para fobias a animales pequeños, al mismo tiempo que analiza los registros fisiológicos del paciente en tiempo real para determinar su estado emocional durante la terapia.

En este estudio se propone evaluar casos de estudios en donde el asistente virtual permite guiar de manera gradual un tratamiento de exposición para fobias a las ratas, tomando ventaja de dispositivos inteligentes para el monitoreo del paciente y ser considerados para determinar el avance del tratamiento.

La terapia propuesta está diseñada con base a la literatura [Ruiz & Valero, 2017; Flobak et al., 2019; Morán et al., 2019; Botella et al., 2000; Campos et al., 2019] adaptada para integrar a VTA, teniendo como metas el aumentar la accesibilidad, motivación y aceptación de las TE para fobias a las ratas.

6. Introducción

La fobia específica es un trastorno mental común de tipo emocional, cognitivo y conductual que afecta a la sociedad. De acuerdo al organismo de Información Científica y Tecnológica para el Congreso de la Unión (INCyTU) se reporta que el 7% de la población padece de una fobia específica, y esta inicia a una temprana edad de 9 años aproximadamente [Rentería-Rodríguez, 2018]. La fobia específica produce un miedo excesivo e irracional hacia objetos o situaciones en particular, donde su exposición ya sea físico o mental con el estímulo fóbico puede provocar un ataque de pánico y ansiedad manifestando diferentes síntomas físicos como son: falta de aliento, sudoración, aceleración del ritmo cardíaco, dolor en el pecho o sensación de asfixia, y/o alteración de conducta con tal de eludir el objeto o situación que provoca la fobia [Gilbert, 2002].

Entre las fobias específicas, se encuentran las fobias hacia los animales pequeños: arácnidos, insectos, roedores y reptiles son los más comunes. Se ha encontrado que los animales pequeños llegan a ser temidos por sus movimientos repentinos, su apariencia física, los sonidos que emiten y sus propiedades táctiles. Generalmente, las personas que sufren estos tipos de fobias tratan de anticiparse al problema evitando exponerse, repercutiendo en las actividades cotidianas en diferentes ámbitos: laboral, académico y social [Bados, 2017].

Actualmente, uno de los tratamientos con mayor efectividad para superar las fobias específicas es una terapia de forma conductual, donde el paciente se expone ante su miedo repetidamente y de forma gradual. El objetivo de la terapia se centra en desensibilizar al paciente, recreando distintas situaciones que provocan la activación de las reacciones de la fobia. A este tipo de terapia se le conoce como Terapia de Exposición (TE). Los componentes que constituyen una TE son la participación consensuada del paciente, el terapeuta y la presencia del objeto de miedo, donde los tres son esenciales para la efectividad de la terapia.

7. Planteamiento del problema

La problemática que existe en emplear TE para fobias a animales pequeños es que no se puede asegurar una exposición gradual, debido a que es complicado controlar el comportamiento del animal temido, más si es un tipo de animal no domesticable, creando la posibilidad de agravar más la fobia del paciente. Esta posibilidad hace que las TE para fobias a animales pequeños tengan una tasa baja de aceptación y una tasa alta de abandono [Gilbert, 2002]. También, existen otros tipos de aspectos a considerar que hace que entre el 60% y el 80% de las personas que padecen una fobia no buscan tratamiento [Wrzesien et al., 2015], esto puede ser problema derivado de la disponibilidad de expertos, traslados de larga distancia, salud, situaciones económicas y sociales [Bados & García, 2011]

Se ha buscado mejorar las TE para fobias a animales pequeños incorporando diferentes tipos de tecnologías, principalmente en TE simbólicas donde el paciente tiene contacto en una situación u objeto fóbico no real, empleando representaciones u abstracciones a través de diferentes tipos de elementos como: visuales, audios, audiovisuales, juegos y simulaciones recreando situaciones u objetos fóbicos virtuales [Ruiz et al., 2012]. Las TE simbólicas permiten recrear y controlar entornos (Horváthová et al., 2016; Miloff et al., 2019) y objetos fóbicos no reales (Botella et al., 2011; Botella et al., 2016; Wrzesien et al., 2015), brindando al paciente confianza y seguridad, motivandolo a enfrentar su miedo. Mientras, que la incorporación de tecnologías de la información como aplicaciones de autoayuda a través de Internet permite mayor accesibilidad a diferentes tratamientos psicológicos, aliviando los problemas antes señalados. Ejemplos de desarrollos de aplicaciones que contribuyen en tratamientos para fobias específicas empleando medios de comunicación virtual por Internet son [Donker et al., 2019; Müller et al., 2011; Hnoohom & Nateeraitaiwa, 2017; Andersson et al., 2009]. Sin embargo, se han detectado áreas de oportunidad

a que su vez podrían verse como características deseables a añadir a los sistemas que apoyan en las TE, que se mencionan a continuación:

A. Sistema centrado en el usuario
   a. Un tratamiento de exposición personalizado según la intensidad de severidad de la fobia y síntomas manifestados por el usuario.
   b. Análisis de datos del contexto del paciente que podrían ser de utilidad para determinar el avance adecuado del tratamiento según el progreso adquirido [Arquissandas et al., 2019].
B. TE autoaplicada a través de un asistente virtual
   a. Un sistema que permite llevar a cabo un tratamiento de exposición para fobias a animales pequeños a través de una interacción verbal, tanto oral como textual y utilizando elementos de apoyo audiovisual.

En este sentido, la propuesta de este trabajo consiste evaluar un asistente terapéutico virtual que sea capaz de guiar al paciente a través de un tratamiento de exposición gradual autoaplicada para fobias a animales pequeños, utilizando: cómputo consciente del contexto y distintos tipos de elementos de apoyo como: imágenes, videos, juegos interactivos y videos inmersivo al usar escenas reales mediante el uso de videos 360º, aplicando sesiones de manera remota. Bajo este esquema, la TE gradual mediante el uso de multimedia interactiva con Thera busca atraer y mantener el interés del usuario mediante sesiones adaptables y aumentar la accesibilidad de tratamientos autoaplicados para superar fobias a animales pequeños [Botella et al., 2000; Campos et al., 2019].

8. Objetivos del proyecto de intervención

Evaluar casos de estudios en donde un asistente inteligente permite guiar de manera gradual un tratamiento de exposición para fobias a las ratas, tomando ventaja de dispositivos inteligentes para el monitoreo del paciente y ser considerados en el progreso del tratamiento y en la intensidad del estímulo fóbico. El protocolo está basado en la literatura [Ruiz & Valero, 2017; Flobak et al., 2019; Morán et al., 2019; Botella et al., 2000; Campos et al., 2019] adaptada para integrar a Thera, teniendo como metas el aumentar la accesibilidad, motivación y aceptación de las TE para fobias a animales pequeños.

9. Marco referencial

Terapias de Exposición (TE)

Las personas con fobias específicas como fobias a animales pequeños, suelen tener miedo o disgusto provocados por experiencias traumáticas, información errónea, observación de conductas de otras personas cercanas o solo por una sensación de amenaza, haciendo que la persona con fobia evite una confrontación con el animal temido [Abramowitz *et al.,* 2019]. El evadir una confrontación con el animal temido es uno de los principales factores que mantiene la fobia y hasta puede provocar incrementar la intensidad con el paso del tiempo. Por esta razón, las terapias de exposición son una de las más eficientes, puesto que su objetivo es exponer al paciente, en situaciones u objetos temidos, a través de técnicas que permiten al paciente una exposición gradual o intensa. La finalidad de de las TE es:

- eliminar creencias o ideas negativas,
- manejar las respuestas emocionales,
- reducir y/o eliminar alteraciones fisiológicas,
- prescindir del uso de comportamientos de seguridad
- e inhibir situaciones temidas.

Tipos y técnicas de terapias de exposición

Existen diferentes tipos de terapias de exposición que pueden ser utilizadas dependiendo de la intensidad de la fobia, distintos contextos y objetivos. [Bados y García, 2011] menciona siete tipos de terapias de exposición, que a su vez [Ruiz et al., 2012] las engloba en dos categorías de terapias:

1) **TE en Vivo**, la cual consiste en que el paciente tenga contacto real directo con una situación u objeto temido, ya sea de forma gradual o inundación, es decir, poniendo al paciente en una situación de alto nivel de ansiedad.

2) **TE simbólicas** donde el paciente tiene contacto en una situación u objeto temido pero no real, sino más bien a través de representaciones u abstracciones. Se puede usar la imaginación, elementos audiovisuales (dibujos, fotos, videos, grabaciones), juegos, simulacros de situaciones temidas o sustitución del objeto temido. Muchas de estas representaciones son logradas con ayuda tecnológica como la realidad virtual y la realidad aumentada.

Existen varias técnicas prácticas que se han desarrollado para la superación de fobias y que pueden ser implementadas dentro de una TE. Las técnicas más usadas son las siguientes [Shunnaq & Raeder, 2016]:

- **Terapia de desensibilización sistemática:** técnica de exposición con el objetivo de exponer al paciente ante su miedo de manera gradual, ante el estímulo fóbico. Esta técnica se centra inicialmente en la aceptación del paciente al tratamiento. Posteriormente se establecen niveles de exposición gradual ante el objeto de miedo de acuerdo al paciente, donde el nivel más bajo es inofensivo y el nivel más alto es una representación donde se obtiene una respuesta de miedo máximo. Y finalmente, el paciente estando tranquilo y relajado, se expone gradualmente al objeto de miedo desde el nivel más bajo hasta el más alto establecido por el terapeuta. El avance de cada nivel depende de la aceptación del paciente, es decir, a que éste se acostumbre y soporte el nivel anterior de forma apacible.
- **Terapia de inundación e implosión:** técnica de exposición donde al paciente se le presenta el nivel más alto del objeto de miedo, generalmente ante una situación real. Algunas veces es difícil definir exactamente una situación porque puede ser reemplazada por imágenes, sonidos, videos, realidad virtual u otras que el terapeuta considere adecuadas. Al mismo tiempo, cuando el paciente se encuentre ante el nivel de miedo, se le solicita que informe de forma detallada lo que experimenta para conocer los orígenes del miedo y desinhibirse.
- **Terapia interoceptiva:** es una técnica común implementada en combinación con otras técnicas de exposición antes mencionadas. Esta terapia tiene la finalidad de controlar los síntomas físicos producidos por la fobia implementando técnicas de respiración o otras técnicas similares. La técnica tiene la finalidad de tranquilizar y relajar al paciente ante el objeto de miedo.

Existen diferentes problemáticas en las TE para fobias a animales. Y es que a pesar de ser uno de los tratamientos más efectivos, tienen una tasa baja de aceptación y una tasa alta de abandono. Debido a que las TE son efectivas por la exposición gradual ante los objetos de miedo, sin embargo, al momento de enfrentarse ante un animal, especialmente si este es una animal no domesticable, no se puede asegurar una exposición gradual y existe la posibilidad de agravar la fobia del paciente y/o lastimarse físicamente al momento de rehuir ante la situación de miedo.

Buscando aliviar el problema antes mencionado, se ha recurrido al desarrollo de tecnología que busca engañar fácilmente los sentidos humanos con el uso de tecnologías inmersivas, generando aplicaciones que sirven como herramientas de apoyo en las TE. Las ventajas de estas aplicaciones, es que permiten construir un entorno controlable y tienen la capacidad de recrear y sustituir al objeto de miedo real. Este tipo de entorno le brinda al paciente mayor confianza y seguridad para enfrentarse a su miedo, aumentando la tasa de aceptación y bajando la tasa de abandono en las TE.

A continuación se detallan algunas aplicaciones tecnológicas que apoyan en las TE:

Exposición Basada en Computadora (CBE)
El uso de la computadora para llevar a cabo una TE para fobias permite exponer al usuario diferentes tipos de elementos como visuales, audio, audiovisuales con contenido del estímulo fóbico. CBE permite llevar a cabo tratamientos de autoayuda, brindan un mayor acceso al tratamiento y minimiza la participación de un terapeuta.

A continuación se muestra algunos trabajos que emplean terapia de exposición basa en computadora:
(Müller et al., 2011). Realizaron un estudio para evaluar la efectividad de un tratamiento de una sesión basado en computadora para reducir el miedo y el comportamiento de evasión hacia las arañas, exponiendo a los pacientes a

imágenes de arañas comparando con imágenes neutrales. El estudio demostró en comparación con un grupo de control, que la terapia CBE tuvo una mayor reducción en el miedo antes y después del tratamiento, tanto en el nivel subjetivo como en el nivel conductual.

(Andersson et al., 2009). Realizaron un estudio donde compararon un tratamiento de autoayuda guiada por internet con un tratamiento de una sesión en vivo en una muestra de pacientes con fobia a las arañas. El tratamiento en Internet consistió en cinco módulos de texto semanales, que se presentaron en una página web, un video en el que se modeló la exposición y se brindó apoyo vía Internet. Los resultados mostraron que los grupos no difirieron en el postratamiento o el seguimiento, concluyendo que el tratamiento de exposición guiado por Internet es un nuevo enfoque prometedor en el tratamiento de la fobia a las arañas.

(Watson et al., 2019). Los autores comprobaron la hipótesis de que la información interoceptiva relacionada con la excitación cardiovascular puede influir en los resultados de la terapia de exposición computarizada para la fobia a las arañas. La TE consistió en que los participantes observarán cuatro bloques de imágenes de arañas. Cada bloque contenía 40 imágenes (35 de arañas y 5 formas abstractas). Seleccionadas aleatoriamente de un banco de 220 imágenes de arañas, que representan una selección de arañas del mundo. Concluyendo que la información interoceptiva si influye en el beneficio de la exposición.

Asistentes Terapeuta Virtual

El desarrollo de tareas tecnológicas como el reconocimiento de voz y habla ha permitido la creación y uso de asistentes virtuales como Alexa, Siri, Cortana, entre otros. Estos asistentes virtuales que nos apoyan en tareas rutinarias simples minimizando tiempo y esfuerzo, han dado entrada a nuevas propuestas de asistentes virtuales especializadas en un ámbito, como es en el área de salud clínica. A continuación se muestra algunos trabajos:

(Wang et al., 2013). Propusieron a EuTalk™ que es un terapeuta virtual y un asistente del habla desarrollado para ayudar a las personas con discapacidades de comunicación mediante el apoyo a las necesidades de comunicación, brindando regímenes de rehabilitación altamente accesibles.

(Wang et al., 2018). Publicaron otro estudio donde utilizaron a EuTalk™, concluyendo que todos los participantes coincidieron en que la app que incluía EuTalk™ establecía un protocolo de tratamiento alternativo utilizable para la rehabilitación de la comunicación.

(Buinhas et al., 2019). Otro estudio con el objetivo de aumentar la adherencia a la medicación, actividad física , dieta, y ajustar la intervención a las necesidades de los usuarios y sus características, emplearon un asistente virtual capaz de hablar y expresar emociones a través de animaciones faciales y corporales. Validada por enfermeras académicas con experiencia en atención primaria, se obtuvo opiniones positivas y sugerencias de mejora.

Otros estudios que emplean asistentes virtuales en el sector de salud han demostrado beneficios como la adherencia a los antidiabéticos orales en personas mayores **(Félix et al., 2019)**, o ayudar a los pacientes con demencia y pérdida de memoria a identificar y recordar recuerdos críticos **(Tiong, et al., 2018)**, y practicar ejercicios de reconstrucción cognitiva para disminuir la ansiedad de hablar en público **(Wang, et al., 2020)**.

10. Fase I: Principales resultados del diagnóstico situacional

Propiedad Intelectual

Los investigadores involucrados en este estudio acuerdan compartir la propiedad intelectual de la aplicación desarrollada, misma que será compartida con la Universidad Autónoma de Baja California, quien ha proporcionado los recursos necesarios para su desarrollo. Además, los trabajos de investigación que sean sometidos a revistas y/o congresos tendrán como coautores a los investigadores involucrados y el orden de autoría se definirá respecto a la contribución específica del documento publicado. En todos los casos, se dará preferencia a la investigadora principal del trabajo de esta tesis doctoral, para aparecer como primer autor, siempre y cuando su contribución así lo amerite.

11. Fase II: Planeación

Diseño de la evaluación

Planteamos realizar una evaluación con grupos independientes, i.e., Randomized Control Trial (RCT). El RCT es considerado la “prueba de oro estándar” para evaluar la eficacia de las intervenciones (McGovern, 2006). El RCT es un tipo de estudio en donde los participantes son asignados al azar a uno de dos o más grupos de intervenciones clínicas. Cada grupo participará en una de las siguientes condiciones (Cada grupo consistirá de 10 sujetos):

- Grupo 1: Grupo de control o lista de espera, los participantes de este grupo no recibirán el tratamiento de inmediato. Se obtendrán las mediciones de los participantes por primera vez y después de un mes una segunda vez. Con el propósito de comparar si hubo cambios al no recibir ninguna intervención.
- Grupo 2. Grupo con intervención con tratamiento de exposición autoaplicada para fobias a las ratas guiado por Thera. Los participantes de este grupo contarán como máximo un mes para llevar a cabo el tratamiento el cual deben superar cuatro etapas de exposición gradual, tomando mediciones antes y después del tratamiento para su comparación.

La evaluación consistirá de las siguientes fases:

1. Reclutamiento y selección de participantes

Los participantes de este estudio serán personas adultas de acuerdo a las leyes mexicanas y que se ofrezcan como voluntarios a participar en él. Se publicará la realización del estudio en diferentes medios como son redes sociales (facebook, whatsapp y twitter) y medios de comunicación de las instituciones de los investigadores participantes en este estudio para su difusión y llegar a más participantes potenciales. Los participantes potenciales podrán contactarse por correo electrónico, whatsapp o llamada telefónica con los encargados de reclutamiento del estudio. Cualquier persona mexicana que comprenda el español puede beneficiarse con la intervención, siempre y cuando cumpla con los siguientes criterios de inclusión y exclusión.

Los criterios considerados para la selección de los participantes son los siguientes:

- Criterios de inclusión
  - Hombres y mujeres de 18 a 60 años.
  - Cumplir con los criterios diagnósticos de fobia específica hacia las ratas (con un grado leve y moderado), obtenida por la estructura de entrevistas para trastornos de ansiedad, en inglés Anxiety Disorder Interview Schedule (ADIS) de DSM-IV [Brown and Barlow, 2014].
  - Tener conocimientos y habilidades digitales básicas (manejo de la computadora e internet).
  - Contar con los dispositivos tecnológicos básicos: correo electrónico, computadora/laptop, smartphone, micrófono, conexión a internet y bluetooth.
- Criterios de exclusión
  - Recibir otro tipo de tratamiento psicológico o psicofarmacológico.
  - Estar diagnosticado con otro tipo de trastorno de ansiedad o alguna psicopatología.
  - Presentar alguna condición médica que ponga en riesgo su vida (enfermedad cardiaca, respiratoria, embarazo, entre otros)

Para verificar que estos criterios se cumplan se aplicará una entrevista estructurada ADIS-IV por videoconferencia [Brown et al., 1994]

2. Previa a la intervención

a. Se solicita firma de formato de consentimiento informado a cada uno de los participantes. El contenido del formato consta en informar al participante las instrucciones, lugar, hora y duración aproximada de la intervención. También, informar los riesgos e inquietudes implicados, beneficios, información que quedará grabada y la protección de dicha información que nos comparta durante en la intervención.

   Con el formato de consentimiento firmado del participante, la información recabada durante el estudio, generará un expediente personal. Todo expediente será almacenado de acuerdo a condiciones éticas con completo respaldo y sólo la investigadora principal tendrá acceso al nombre completo, mientras que los investigadores asociados tendrán acceso a los resultados obtenidos más no a datos de identificación. Los expedientes serán mantenidos en completa protección durante un lapso mínimo de cinco años, se aclara que las bases de datos con los puntajes obtenidos en los instrumentos no cuentan con datos de identificación. Y toda información presentada en foros de discusión mantendrán resultados globales de promedios, mas no puntajes individuales ni datos de identificación personal.

b. Se contacta con el participante y se le invita acudir al lugar específico para asistir a la intervención en donde se contará con los dispositivos necesarios para implementar el tratamiento. Los dispositivos son: (1) *SmartWatch*, utilizado especialmente para la adquisición de la frecuencia cardíaca en tiempo real del participante; (2) *Aplicación VTA,* que permite una interacción verbal del participante para determinar el progreso de la sesión de la TE; (3) dispositivo (PC, laptop, tablet) con: aplicación *VTA* y modelo de detección ocular, conexión a internet, cámara y micrófono para observar y escuchar el comportamiento del participante y conexión *bluetooth* para establecer la comunicación entre los dispositivos; y, (4) *Smartphone* y Google *Cardboard* para la inmersión del video 360º.
c. Se le instruye al participante sobre el espacio de la intervención: colocarse frente al dispositivo y acceder a la aplicación VTA donde el participante pueda escuchar y responder, colocarse el *SmartWatch* y sincronizarlo para la obtención del ritmo cardiaco.
d. Se establece la línea base del participante, obteniendo las medidas previas: miedo y evitación a las ratas, lectura del ritmo cardiaco y detección ocular.

3. Intervención

A. *VTA* ubicada en el equipo de cómputo local, inicia sincronizando la comunicación entre los dispositivos *SmartWatch, cámara y micrófono.* En caso de que falle la sincronización o haya algún otro problema técnico, VTA deberá proporcionar información para soporte técnico y/o instrucciones de cómo resolver el problema.

B. Posteriormente, *VTA* crea el registro del participante que por medio de llenado de formularios permite conocer qué tan familiarizado se encuentra el participante interactuar con un asistente virtual y obtener datos generales del usuario e información base como:
a. Nivel de miedo a las ratas por medio del cuestionario (FRQ: siglas en inglés “Questionnaire on the fear of rats”). Cuestionario adaptado de (FSQ; Fear of Spider Questionnaire; Szymanski & O'Donohue, 1995).
b. Nivel de ansiedad a las ratas por medio de *Inventario de ansiedad rasgo-estado (IDARE; Díaz-Guerrero & Spielberger, 1975)*.
c. *Otros miedos por medio del cuestionario de miedo (FQ; Fear Questionnaire; Marks & Mathews, 1979)*.

En caso de que el participante ya se encuentre registrado, obtiene información de su avance como el número de sesiones, reacciones fisiológicas durante su exposición, progreso y avance de la terapia. Con el valor de la severidad de la fobia se determina el nivel de exposición del objeto fóbico que iniciará el participante.

C. En caso de que el participante no se encuentre familiarizado con un asistente virtual, se le proporcionará una etapa de entrenamiento para mostrar cómo funciona y lo que puede realizar, proporcionando todas las posibles instrucciones que puede solicitar a VTA. Con el propósito de que el participante sienta la confianza de ser escuchado y que tiene control del avance del programa y ser asistido en cualquier momento. Por lo que se requiere que el participante se encuentre decidido a enfrentar su fobias con tal de mejorar su calidad de vida, reduciendo los malestares provocados por ello.

D. Iniciando la sesión*, VTA* le da la bienvenida al participante y le informa sobre el contenido y el objetivo del tratamiento de exposición para fobias a las ratas.

E. *VTA* clasifica la frecuencia cardíaca a través de la lectura de los pulso por minuto (ppm) proporcionados por el *SmartWatch*, para evaluar y determinar cuatro niveles diferentes: 1) **Normal**, el ritmo cardíaco se encuentra dentro del rango normal establecido e indica la continuidad de la sesión; 2) **Alterado bajo**: ritmo cardíaco entre el umbral A y umbral B. Indica la presencia de una alteración fisiológica leve donde el participante aún puede tolerar la situación y habituarse a ella. 3) **Alterado alto**: ritmo cardiaco entre el umbral B y la frecuencia cardiaca máxima. Indica una alteración fisiológica alta, la terapia de exposición se detiene por un momento y VTA propone empezar a realizar un ejercicio de respiración profunda como estrategia de relajación para calmar al participante y cambiar su estado a alterado bajo o normal; por último, 4) **Crisis**: el ritmo cardíaco rebasa el rango máximo. VTA trata de reducir la actividad cardiaca a través del ejercicio de respiración profunda y existe la posibilidad de que el participante decida finalizar la sesión del día. En las Tablas 1 y 2 se muestran las

variables empleadas y los rangos establecidos para determinar las distintas categorías del estado del participante [American Heart Association, 2020].

Tabla 1. Variables consideradas en la frecuencia cardíaca.

Variables	Valor
Edad	Edad del usuario
Frecuencia Cardíaca Máxima (FCM)	*FCM* = 220 - Edad
Umbral A	A= (*FCM* * 0.50) ppm
Umbral B	*B*= (*FCM* * 0.85) ppm

Tabla 2. Clasificación del estado de la frecuencia cardiaca del usuario.

Estados	Frec. Cardíaca
**Crisis**	Mayor *FCM*
**Alterado alto**	Entre *B* a *FCM*
**Alterado bajo**	Entre *A* a *B* y menor de 60 ó 40 ppm
**Normal**	Si realiza ejercicio: entre 40 ppm a *A*<br>No realiza ejercicio: entre 60 ppm a *A*

F. VTA le pregunta al usuario si se encuentra listo para iniciar la terapia de exposición virtual del objeto fóbico. Sí VTA clasifica la frecuencia cardiaca del usuario en estado de alteración alta iniciará un ejercicio de respiración profunda hasta que se haya tranquilizado, es decir que se encuentre en un nivel normal. Si obtiene una contestación negativa o no recibe una contestación a la pregunta cuando no se detecta una alteración, esperará hasta que el participante se haya preparado poniendo de fondo música relajante. De no existir ninguna de las dos condiciones anteriores, se continuará con la sesión establecida.

G. En esta parte de la sesión el participante se expone al objeto fóbico. La exposición está dividida en cuatro etapas considerando una exposición gradual con base al realismo, interacción e intensidad del objeto según la taxonomía propuesto por Morán y colaboradores [Morán et al., 2020].

- La primera y segunda etapa, el participante es expuesto al objeto fóbico apoyado con material multimedia basados en el instrumento *Multimedia Behavioural Avoidance Test* (MBAT). El MBAT es una prueba reactiva donde se presentan al participante diversos estímulos visuales (imágenes y videos) relacionados con el objeto de miedo [Ruiz & Valero, 2017].
- En la tercera etapa, el participante interactúa de manera virtual con representaciones de ratas, utilizando varios niveles de exposición gradual en un videojuego [Hnoohom & Nateeraitaiwa, 2017; Shunnaq & Raeder, 2016].
- Por último, la cuarta etapa el participante a través de videos 360º, empleando *smartphone* y google *cardboard* (elementos accesibles), es sumergido en un ambiente de realidad virtual simulando una situación natural (como ejemplo ver página Web Covid feel good, 2020), en donde el contenido del video se mostrarán ratas con distintos tipos de comportamiento naturales.

Las etapas del tratamiento serán escalables, es decir, el participante deberá superar una etapa para avanzar a la siguiente. Sin embargo, VTA puede desbloquear algunas de las primeras etapas, comparando el grado de severidad del participante con el grado de severidad establecida en cada etapa del tratamiento.

El contenido de cada etapa fue retroalimentado y validado por especialistas en intervención a fobias. A continuación se describe a detalle cada etapa.

a. Primera etapa: exposición gradual a través de imágenes

En caso de que el participante tenga una fobia de severidad moderada se aconseja que la exposición inicie de forma visual a través de imágenes. El propósito de esta etapa es que el participante pueda habituarse y tolerar el aspecto de las ratas, a través de 10 imágenes donde se observa cada una de las ratas al menos 10 segundos, procedimiento adaptado de Ruiz & Valero [2020].

A continuación se detalla el proceso del tratamiento en la primera etapa:

a. VTA informa al participante el contenido y el objetivo de la primera etapa.
b. VTA le informa al participante que le mostrará una imagen, le describe el contenido y le instruye que es necesario que la observe durante 10 seg.
c. VTA le pregunta al participante, si se encuentra listo para observar la imagen.
d. En caso que el participante conteste negativamente, VTA le comenta que le dará 1 minuto para alistarse y volverá a preguntar. De lo contrario, VTA le mostrará la imagen correspondiente.
e. VTA clasifica la frecuencia cardiaca del participante (proceso explicado anteriormente en el punto 8).
f. Al transcurrir los 10 seg., si el participante no mostró una alteración en su frecuencia cardíaca y observó sin vacilaciones la pantalla, VTA continuará con la siguiente imagen. De lo contrario, VTA le informará que le mostrará de nuevo la misma imagen hasta que se habitúe y soporte observar la imagen.
g. Se repiten los pasos del b al f, hasta que el participante pueda observar sin alterarse, las diez imágenes descritas en la Tabla 3.
h. En la mitad (después de la quinta imagen) y al terminar la primera etapa, VTA le preguntará al participante que conteste con la mayor sinceridad las siguientes preguntas: utilizando una escala del 1 al 10 donde 1 es nada y 10 es mucho, ¿cuánto considera que pudo observar las ratas en las imágenes? y ¿cuál es la intensidad de ansiedad que considera que haya experimentado con las imágenes mostradas?

Tabla 3. Contenido de las imágenes que se mostrarán en la etapa 1.

	**Realismo**	**Intensidad**			**Interacción visual**
**Imagen**	**Tipo de imagen**	**Cantidad de ratas**	**Tamaño**	**Apariencia**	**Observar contenido**
1	Dibujo caricatura	1	pequeña	agradable	una rata sola
2	Dibujo realista	1	mediana	inofensiva	una rata sola
3	Foto	1	pequeña	inofensiva	una rata sola
4	Foto	1	mediana	inofensiva	una rata sobre la mano de una persona
5	Foto	1	mediana	inofensiva	una rata sola
6	Foto	1	grande	inofensiva	una rata sobre el hombro de una persona
7	Foto	1	grande	no agradable	hambrienta
8	Foto	3	grandes	no agradables	rata con crías
9	Foto	más de 4	grandes	no agradables	entre la basura
10	Foto	muchas	grandes	no agradables	colonia de ratas

b. Segunda etapa: exposición gradual a través de videos

En caso de que el participante tenga una fobia de severidad moderada a leve, se aconseja realizar una exposición de forma audiovisual a través de videos. El propósito de esta etapa es que el participante pueda habituarse y tolerar el aspecto y comportamiento natural de las ratas a través de la observación de 6 videos cortos con una duración de 1 minuto según el video, procedimiento adaptado de Ruiz y Valero [2020].

A continuación se detalla el proceso del tratamiento en la segunda etapa:

a. VTA informa al participante el contenido y el objetivo de la segunda etapa.
b. VTA le informa al participante que le mostrará un video, le describe el contenido y su duración, y que es necesario que lo observe en todo momento.
c. VTA le pregunta al participante, si se encuentra listo para observar el video.
d. En caso que el participante conteste negativamente, VTA le comenta que le dará 1 minuto para alistarse y volverá a preguntar. De lo contrario, VTA le mostrará el video correspondiente.
e. VTA valora el estado emocional del participante (proceso explicado anteriormente en el punto 8).
f. Si el participante no mostró una alteración en su frecuencia cardíaca y observó la pantalla en lo que duró transmitiendo el video, VTA continuará con el siguiente video. De lo contrario VTA le informará que le mostrará de nuevo el mismo video, hasta que se habitúe y soporte observar todo el video.
a. Se repiten los pasos b al f, hasta que el participante pueda observar sin alterarse los seis videos cortos descritos en la Tabla 4.
b. En la mitad (después del tercer video) y al terminar la segunda etapa, VTA le preguntará al participante que conteste con la mayor sinceridad las siguientes preguntas: utilizando una escala del 1 al 10 donde 1 es nada y 10 es mucho, ¿cuánto considera que pudo observar las ratas? y ¿cuál es la intensidad de ansiedad que considera que haya experimentado con los videos mostrados?

Tabla 4. Contenido de los videos que se mostrarán en la etapa 2.

	**Intensidad**	**Interacción**	**Realismo**
**Video**	**Cantidad**	**Observar contenido del video**	**Aspecto**
1	1	Cómo dibujar una rata	dibujo
2	1	Rata amigable	caricatura infantil
3	1	Rata y su jaula	real
4	más de 1	Rata con sus cría y su cuidadora	real
5	más de 1	Comunidad de ratas	caricatura realista
6	más de 1	Comunidad de ratas callejeras	reales

c. Tercera etapa: Interacción gradual con ratas virtuales.

En caso de que el participante tenga una fobia de severidad leve a las ratas, la exposición gradual puede llevarse a cabo a través de un juego serio que emplee ratas virtuales. El propósito de la etapa del videojuego es que el participante pueda acercarse, observar e interactuar con distintas ratas virtuales mediante un videojuego en primera persona, en donde los movimientos del participante son dirigidos a través del teclado de la PC. Como ejemplo, en la Figura 1 se muestra una escena virtual, donde el participante se encuentra interactuando con ratas en primera persona. Algunos trabajos relacionados han mostrado que los juegos son efectivos en tratamientos de otro tipos de fobias específicas, como perros o arañas [Hnoohom & Nateeraitaiwa, 2017; Shunnaq & Raeder, 2016].

El videojuego consta de cinco niveles que incrementa la intensidad de exposición a través del aspecto y tamaño de la rata virtual, como se muestra en la Tabla 5. El escenario del juego es en una casa que recientemente se ha adquirido, donde el participante deberá explorar y encontrar ratas a las que tendrá que deshacerse para habitar su nueva casa. Durante la exploración de la casa, el participante encontrará distintas ratas que activarán

minijuegos con modelos de ratas 3D para adquirir artículos que ayudarán posteriormente a capturarlas. La superación de los minijuegos incrementará el tamaño e intensidad de velocidad y movimientos de las ratas. El participante al ir capturando cierta cantidad de las ratas, será recompensado mejorando y desbloqueando otros espacios de su casa .

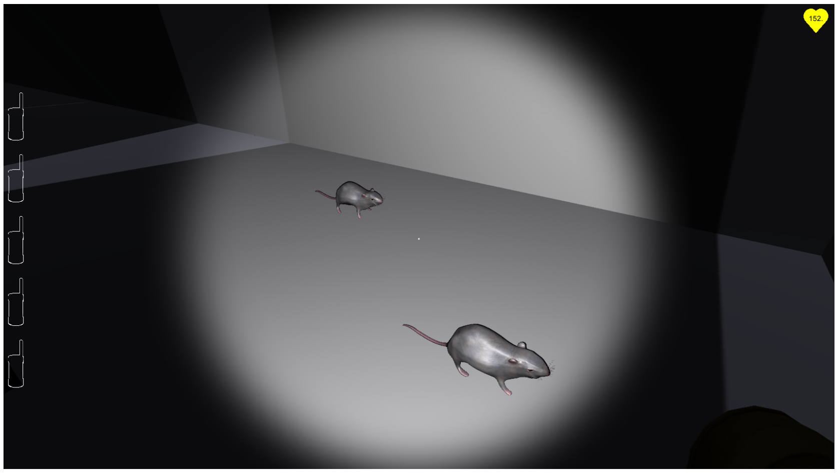

Figura 1. Ejemplo de interacción virtual con ratas, con impresión de primera persona.

Tabla 5. Aspecto y tamaño de las ratas virtuales según el nivel de exposición del videojuego.

Nivel	Aspecto	Tamaño
1	animado	pequeñas
2	animado	medianas
3	real	pequeñas
4	real	medianas
5	real	grandes

A continuación se detalla el proceso del tratamiento en la tercera etapa:

a. VTA le informa al participante que el objetivo principal de la etapa es poder lograr que pueda soportar y enfrentar situaciones temidas con ratas expuestas a través de un videojuego. Posteriormente, le informa sobre el contexto e instrucciones necesarias para poder realizar el juego, por ejemplo cómo desplazarse y recoger objetos en el juego. También, le explicará que al lado izquierdo de la pantalla se irán activando artículosque vaya encontrando y al lado derecho superior de la pantalla, se mostrará un corazón donde su color cambiará según su estado emocional: verde, si se encuentra tranquilo o normal; amarillo, si se encuentra alterado bajo; naranja, si se encuentra alterado alto; y rojo, si se encuentra en crisis.
b. Sí VTA detecta al participante normal o solo un poco alterado (alterado bajo), VTA le pregunta si desea iniciar el juego.
c. Si la contestación del participante es positiva, VTA empieza el juego, pero si la contestación es negativa o no se obtuvo una contestación, VTA esperará 15 seg para realizar de nuevo la pregunta. En caso de que VTA haya preguntado tres veces y no obtenga una contestación positiva, VTA preguntará si desea continuar con la sesión, a lo que si el participante no contesta de manera positiva, VTA terminará la sesión.
d. Empieza el juego y el participante aparece en la puerta principal de la casa.
e. VTA le instruye al participante que la única manera de habitar su nueva casa es deshaciéndose de las ratas, y para ello es necesario de armarse de valor y explorar los espacios de la casa.
f. Cuando el participante se encuentre recorriendo los espacios desbloqueados de la casa, se encontrará con distintas ratas que a medida que el participante vaya encontrando y capturando las ratas, la exposición de ratas irá incrementando los movimientos, desplazamiento, cantidad y frecuencia de aparición. En la Tabla 6 se

muestra el comportamiento e interacción gradual de las ratas según el número de ratas capturadas por el participante.

g. En la mitad (después del tercer nivel) y al terminar el videojuego, VTA le preguntará al participante que conteste con la mayor sinceridad las siguientes preguntas: utilizando una escala del 1 al 10 donde 1 es nada y 10 es mucho, ¿cuánto considera que pudo observar las ratas 3D? y ¿cuál es la intensidad de ansiedad que considera que haya experimentado en el videojuego?

Tabla 6. Comportamiento e interacción gradual de ratas virtuales en el videojuego.

Pieza	Comportamiento e interacción gradual de las ratas virtuales
1	El participante no haya capturado ninguna rata, la frecuencia y velocidad de las ratas dentro del minijuego será lenta de tamaño chicas de aspecto animado.
2	El participante haya capturado 1 a 3, la frecuencia y velocidad de las ratas dentro del minijuego será medio lento con tamaño mediano de aspecto animado.
3	El participante haya capturado 4 a 6, la frecuencia y velocidad de las ratas dentro del minijuego será moderada con tamaño chicas de aspecto real.
4	El participante haya capturado 7 a 9, la frecuencia y velocidad de las ratas dentro del minijuego será moderada con tamaño mediano de aspecto real.
5	El participante haya capturado 10 a 11, la frecuencia y velocidad de las ratas dentro del minijuego será rápida con tamaño grandes de aspecto real.

h. Al momento que el participante captura una rata, VTA lo felicita, verifica la duración del minijuego, si la duración fué menos de 1 minuto y no tuvo alguna alteración fisiológica, le otorga un nuevo artículo para casa. Después, VTA le informa al participante que continúe explorando y. En caso de que el participante haya capturado la última rata se mostrará un video corto en donde el participante puede observar todos los espacios de su casa limpios, remodelados con todos los artículos ganados.

d. Cuarta etapa: Inmersión en un entorno de Realidad Virtual (RV).

En caso de que el participante tenga una fobia de severidad leve a las ratas, pueden realizar una exposición en RV mediante el uso de gafas y un video 360º . Empleando diferentes videos de escenas urbanas o rurales, donde se observa alrededor ratas. El propósito de esta etapa es que el participante pueda sumergirse y poder soportar observar el comportamiento de las ratas callejeras que se encuentran alrededor de la escena, como ejemplo [Flobak et al., 2019] que crearon prototipos de escenarios de realidad virtual para TE, habilitados por video de 360º basados en experiencias vividas.

A continuación se detalla el proceso del tratamiento en la cuarta etapa:

a. VTA informa al participante el contenido y el objetivo de la cuarta etapa.
b. VTA le informa al participante que le mostrará un video de 360º empleando un Google cardboard que permite una inmersión de RV, describe el contenido del video y le instruye que es necesario observar a detalle según la duración del video.
c. VTA valora el estado emocional del participante (proceso explicado anteriormente en el punto 8).
d. Si el participante no mostró una alteración en su frecuencia cardíaca y observó la escena en lo que duró transmitiendo el video, VTA continuará con el siguiente video. De lo contrario VTA le informará que le mostrará de nuevo el mismo video, hasta que se habitúe y soporte observar el video.
e. Se repiten los pasos b, c y d, hasta que el participante pueda observar sin alterarse seis videos cortos descritos en la Tabla 7.
f. Después de observar cada video inmersivo, VTA le preguntará al participante que conteste con la mayor sinceridad las siguientes preguntas: utilizando una escala del 1 al 10 donde 1 es nada y 10 es mucho, ¿cuánto

considera que pudo observar las ratas? y ¿cuál es la intensidad de ansiedad que considera que ha experimentado el video inmersivo?

Tabla 7. Contenido de los videos 360º que se mostrarán en la etapa 4.

Video	Duración	Escena	Observar contenido
1	3 min	Urbana, en lugares concurridos de la ciudad	Mostrando a lo lejos una o varias ratas reales
2	3 min	Dentro del hogar	Aparece una rata real recorriendo en la habitación

I. Al finalizar cada una de las etapas del tratamiento, VTA analizará varios aspectos para determinar si el participante superó la etapa correspondiente. Verificando lo siguiente:

1. **Alteración fisiológica**: durante la etapa correspondiente el participante no haya tenido una *alteración alta* o que la mayor parte de la etapa se encontrará en un nivel *alterado bajo*.
2. **Evitación**: a) el ***tiempo*** que duró la etapa, b) el ***modo de contestar***, ya sea positivamente o negativamente para mostrar los siguientes elementos de exposición (imágenes, videos, niveles del videojuego o videos inmersivos), y c) **unidad subjetiva evitacional**, proporcionado por el participante en la mitad y al terminar cada etapa.
3. **Unidad subjetiva de ansiedad,** proporcionado por el participante en la mitad y al terminar cada etapa.

Sí VTA determina que el participante superó la etapa, iniciará la siguiente o habrá terminado la TE si la etapa superada era la última, pero sí VTA determina que el participante no superó la etapa, repetirá de nuevo la misma.

4. Evaluación

Una vez terminado el tratamiento para fobias a las ratas o de haber pasado los cuatro días de periodo máximo se le solicitará a los participantes completar las siguientes encuestas:

a. *Cuestionario miedo a las ratas (FRQ, Questionnaire on the fear of rats)*: cuestionario adaptado por el Cuestionario miedo a las arañas (FSQ; Fear of Spider Questionnaire; Szymanski & O'Donohue, 1995).

b. *Inventario de ansiedad estado-rasgo (IDARE)*: La versión en español del STAI (State-Trait Anxiety Inventory) consta de 40 enunciados; 20 evalúan ansiedad rasgo y 20, ansiedad estado. Ha sido validado en México por Rojas-Carrasco (2010), obteniendo una confiabilidad adecuada para las dos dimensiones de ansiedad (coeficientes alfa de Cronbach mayores a 0.83).

c. *Cuestionario de miedo (FQ; Fear Questionnaire; Marks & Mathews, 1979)*: evalúa la intensidad con la que se evitan y preocupan determinadas situaciones, y también contiene una escala donde el paciente valora el estado actual de sus síntomas fóbicos.

d. *Sentido de presencia y juicio de realidad:* este instrumento evalúa el grado de presencia experimentado a través de los elementos visuales mediante dos preguntas con una escala Likert de 0 a 10 (0 = nada, 10 = demasiado) (Juan et al., 2005): 1) ¿Hasta qué punto te has sentido presente en la inmersión? y 2) ¿En qué medida te has sentido en un lugar en el que aparecían ratas?.

e. Cuestionario de Satisfacción del Cliente (CSQ-8S, *Client Satisfaction Questionnaire*): consta de 8 preguntas con una puntuación total que oscila entre 8 y 32 para medir el nivel de satisfacción con el tratamiento y se ha utilizado para evaluar tratamientos autoaplicados (Palma-Gomez, et al. , 2020). La pregunta “¿Hasta qué punto lo encontró aversivo?” se añadió a este cuestionario (Botella et al., 2009).

f. *Cuestionario de Usabilidad de Telesalud (TUQ, Telehealth Usability Questionnaire): evalúa la usabilidad de los sistemas de telesalud (Parmato et al. 2016), a través de dos dimensiones: 1) efectividad y 2) facilidad de uso. Contiene 12 preguntas con una escala Likert 7 que va del 1 al 7 (1 = en desacuerdo, 7 = de acuerdo).*

g. *Escala de mejora del paciente CGI-I (Global Clinical Impression Scale, CGI; Guy, 1976): contiene un ítem adaptado de la escala CGI y que permite al paciente evaluar su grado de mejora en una escala de 7 puntos (1 = mucho mejor, 7 = mucho peor).*

12. Protección de información de los participantes

Los participantes serán valorados de manera individual y únicamente el personal capacitado en la aplicación de instrumentos tendrá contacto directo con el/la participante. Serán informados antes de proceder con cualquier aplicación sobre el uso de los datos, la libertad con la que cuentan para abandonar el estudio en caso de desearlo en cualquier momento del proceso, y por último decidirán si desean o no participar en el protocolo dando su autorización o rechazo por escrito al evaluador.

Se realizará un expediente personal de aquellos que participen en la aplicación de las pruebas, únicamente en el consentimiento vendrá especificado su nombre y firma y en el resto del documento se manejara sus iniciales. Todo expediente será almacenado de acuerdo a condiciones éticas con completo respaldo y sólo el investigador principal tendrá acceso a datos confidenciales, mientras que los investigadores asociados tendrán acceso a los resultados obtenidos más no a datos de identificación. Los expedientes serán mantenidos en completa protección durante un lapso mínimo de cinco años, se aclara que las bases de datos con los puntajes obtenidos en los instrumentos no cuentan con datos de identificación. Y toda información presentada en foros de discusión mantendrán resultados globales de promedios, mas no puntajes individuales ni datos de identificación personal.

Los expedientes generados en formato digital serán archivados en una carpeta con permiso de acceso solo al personal autorizado otorgado por el investigador principal. Dichos archivos serán resguardados por al menos 5 años para su posterior destrucción.

13. Referencias bibliográficas

- Abramowitz JS, Deacon BJ, Whiteside SPH. (2019). Exposure Therapy for Anxiety: Principles and Practice. Second Edi. New York: The Guilford Press. www.guilford.com.
- Andersson, G., Waara, J., Jonsson, U., Malmaeus, F., Carlbring, P., & Öst, L. G. (2009). Internet-based self-help versus one-session exposure in the treatment of spider phobia: A randomized controlled trial. Cognitive Behaviour Therapy, 38(2), 114–120. https://doi.org/10.1080/16506070902931326
- Arquissandas, P., Lamas, D., & Oliveira, J. (2019). Augmented Reality and Sensory Technology for Treatment of Anxiety Disorders. *2019 14th Iberian Conference on Information Systems and Technologies (CISTI)*, (June), 1–4. https://doi.org/10.23919/cisti.2019.8760859
- Bados, A. (2017). Fobias Específicas. Barcelona, España.
- Botella, C., Quero, S., Serrano, B., Baños, R. M., & García-Palacios, A. (2009). Avances en los tratamientos psicológicos: La utilización de las nuevas tecnologías de la información y la comunicación [Advances in psychological treatments: The use of new information and communication technologies]. Anuario de Psicología, 40(2), 155–170.
- Botella, C., Breton-López, J., Quero, S., Baños, R. M., García-Palacios, A., Zaragoza, I., & Alcaniz, M. (2011). Treating cockroach phobia using a serious game on a mobile phone and augmented reality exposure: A single case study. *Computers in Human Behavior*, *27*(1), 217–227. https://doi.org/10.1016/j.chb.2010.07.043
- Botella, C., Fernández-Álvarez, J., Guillén, V., García-Palacios, A., & Baños, R. (2017). Recent Progress in Virtual Reality Exposure Therapy for Phobias: A Systematic Review. *Current Psychiatry Reports*, *19*(7). https://doi.org/10.1007/s11920-017-0788-4
- Botella, C., Pérez-Ara, M. Á., Bretón-López, J., Quero, S., García-Palacios, A., & Baños, R. M. (2016). In Vivo versus augmented reality exposure in the treatment of small animal phobia: A randomized controlled trial. *PLoS ONE*, *11*(2), 1–22. https://doi.org/10.1371/journal.pone.0148237
- Botella, C., Baños, R., Guillén, V., Perpiña, C., Alcañiz, M., Pons, A. (2000). Telepsychology: Public Speaking Fear Treatment on the Internet. CyberPsychology & Behavior, 3(6), 959–968. doi:10.1089/109493100452228
- Brown, T. A., & Barlow, D. H. (2014). Anxiety and related disorders interview schedule for DSM-5 (ADIS-5L) : client interview schedule, 114.

- Buinhas, S., Cláudio, A. P., Carmo, M. B., Balsa, J., Cavaco, A., Mendes, A., ... Guerreiro, M. P. (2019). Virtual assistant to improve self-care of older people with type 2 diabetes: First prototype doi:10.1007/978-3-030-16028-9_21
- Campos, D., Bretón-López, J., Botella, C., Mira, A., Castilla, D., Mor, S., Baños, R. & Quero, S. (2019). Efficacy of an internet-based exposure treatment for flying phobia (NO-FEAR Airlines) with and without therapist guidance: a randomized controlled trial. BMC Psychiatry, 19(1), 86–. doi:10.1186/s12888-019-2060-4
- Díaz-Guerrero, R., & Spielberger, C.D. (1975). STAI: Anxiety Inventory: Trait-State. [IDARE: Inventario de ansiedad: rasgo-estado]. El Manual Moderno.
- Donker, T., Cornelisz, I., van Klaveren, C., van Straten, A., Carlbring, P., Cuijpers, P., & van Gelder, J. L. (2019). Effectiveness of Self-guided App-Based Virtual Reality Cognitive Behavior Therapy for Acrophobia: A Randomized Clinical Trial. JAMA psychiatry, 76(7), 682–690. https://doi.org/10.1001/jamapsychiatry.2019.0219
- Félix, I. B., Guerreiro, M. P., Cavaco, A., Cláudio, A. P., Mendes, A., Balsa, J., Carmo, M. B., Pimenta, N., & Henriques, A. (2019). Development of a complex intervention to improve adherence to antidiabetic medication in older people using an anthropomorphic virtual assistant software. Frontiers in Pharmacology, 10, 680 https://doi.org/10.3389/fphar.2019.00680
- Flobak, E., Wake, J. D., Vindenes, J., Kahlon, S., Nordgreen, T., & Guribye, F. (2019). Participatory design of VR scenarios for exposure therapy. Conference on Human Factors in Computing Systems - Proceedings, 1–12. https://doi.org/10.1145/3290605.3300799
- Gilbert, S. (2002). *Coping with Anxiety and Phobias*. Boston. Retrieved from www.health.harvard.edu
- Guy, W. (1976). ECDEU Assessment Manual for Psychopharmacology. Rockville, MD: US Department of Health, Education, and Welfare Public Health Service Alcohol, Drug Abuse, and Mental Health Administration.
- Hnoohom, N., & Nateeraitaiwa, S. (2017). Virtual reality-based smartphone application for animal exposure. 2nd Joint International Conference on Digital Arts, Media and Technology 2017: Digital Economy for Sustainable Growth, ICDAMT 2017, 417–422. https://doi.org/10.1109/ICDAMT.2017.7905004
- Horváthová, D., Siládi, V., & Lackova, E. (2016). Phobia treatment with the help of virtual reality. *2015 IEEE 13th International Scientific Conference on Informatics, INFORMATICS 2015 - Proceedings*, 114–119. https://doi.org/10.1109/Informatics.2015.7377818
- Juan, M. C., Alcañiz, M., Monserrat, C., Botella, C., Baños, R. M., & Guerrero, B. (2005). Using augmented reality to treat phobias. IEEE Computer Graphics and Applications, 25(6), 31–37. https://doi.org/10.1109/MCG.2005.143
- Marks, I. M., & Mathews, A. M. (1979). Brief standard self-rating for phobic patients. Behaviour research and therapy, 17(3), 263–267. https://doi.org/10.1016/0005-7967(79)90041-x
- Miloff, A., Lindner, P., Dafgård, P., Deak, S., Garke, M., Hamilton, W., Carlbring, P. (2019). Automated virtual reality exposure therapy for spider phobia vs. in-vivo one-session treatment: A randomized non-inferiority trial. *Behaviour Research and Therapy*, *118* (June 2018), 130–140. https://doi.org/10.1016/j.brat.2019.04.004
- Morán, A., Ramírez-Fernández C., Meza-Kubo V. (2020). Design of Smart Objects of Fear with a Taxonomy of Factors Affecting the User Experience of Exposure Therapy Systems for Small-Animal Phobias.
- Müller, B. H., Kull, S., Wilhelm, F. H., & Michael, T. (2011). One-session computer-based exposure treatment for spider-fearful individuals - Efficacy of a minimal self-help intervention in a randomised controlled trial. Journal of Behavior Therapy and Experimental Psychiatry, 42(2), 179–184. https://doi.org/10.1016/j.jbtep.2010.12.001
- Palma-Gómez, A., Herrero, R., Banõs, R., Garciá-Palacios, A., Castañeiras, C., Fernandez, G. L, Llull, D. M., Torres, L. C., Barranco, L. A., Cárdenas-Gómez, L., & Botella, C. (2020). Efficacy of a self-applied online program to promote resilience and coping skills in university students in four Spanish-speaking countries: Study protocol for a randomized controlled trial. BMC Psychiatry, 20(1), 1–16. https://doi.org/10.1186/s12888-020-02536-w
- Parmanto, B., Lewis, Jr., A. N., Graham, K. M., & Bertolet, M. H. (2016). Development of the Telehealth Usability Questionnaire (TUQ). International Journal of Telerehabilitation, 8(1), 3–10. https://doi.org/10.5195/ijt.2016.6196
- Rentería Rodríguez, M. E. (2018). *Salud mental en México. NOTA-INCyTU [Internet] 2018 [acceso 22 de enero de 2019]; 007:1-6* (Vol. 52). Ciudad de México. Retrieved from https://www.foroconsultivo.org.mx
- Rojas-Carrasco, K. E. (2010). Validación del Inventario de Ansiedad Rasgo-Estado en padres con un hijo en terapia intensiva. [The state-trait anxiety inventory for parents who have hospitalized children in an intensive care unit] Revista Médica Del Instituto Mexicano Del Seguro Social, 48(5), 491-496.

- Abramowitz, J. S., Deacon, B. J., & Whiteside, S. P. H. (2019). Exposure Therapy for Anxiety: Principles and Practice (Second Edi). New York: The Guilford Press. Retrieved from www.guilford.com
- Ruiz, A., & Valero, L. (2017). Eficacia de un tratamiento de exposición progresiva multimedia para fobias específicas: un estudio clínico y experimental [Efficacy of a treatment of multimedia progressive exposure for specific phobias: An experimental and clinical study] [Doctoral Dissertation, University of Malaga]. https://hdl.handle.net/10630/15534
- Ruiz-García, A., & Valero-Aguayo, L. (2020). Multimedia intervention for specific phobias: A clinical and experimental study. Psicothema, 32(3), 298–306. https://doi.org/10.7334/psicothema2020.87
- Szymanski, J., & O'Donohue, W. (1995). Fear of Spiders Questionnaire. Journal of behavior therapy and experimental psychiatry, 26(1), 31–34. https://doi.org/10.1016/0005-7916(94)00072-t
- Shunnaq, S., & Raeder, M. (2016). VirtualPhobia: A Model for Virtual Therapy of Phobias. *Proceedings - 18th Symposium on Virtual and Augmented Reality, SVR 2016*, 59–63. https://doi.org/10.1109/SVR.2016.20
- Tiong, E. Y. C., Powers, D. M. W., & Maeder, A. J. (2018). Dementia virtual assistant as trainer and therapist: Identifying significant memories and interventions of dementia patients. ACM International Conference Proceeding Series, 1–9. https://doi.org/10.1145/3167918.3167953
- Wang EH, Chen SH, Zhou L, Hill K, (2013) "EuTalk: A Virtual Therapist and Speech Assistant for People with Communication Disabilities", SDA Conference, University of Pittsburgh, Pittsburgh, PA. (October 31, 2013 - November 1, 2013)
- Wang, E. -., Zhou, L., Chen, S. -. K., Hill, K., & Parmanto, B. (2018). Development and evaluation of a mobile AAC: A virtual therapist and speech assistant for people with communication disabilities. Disability and Rehabilitation: Assistive Technology, 13(8), 731-739. doi:10.1080/17483107.2017.1369592
- Wang, J., Yang, H., Shao, R., Abdullah, S., & Sundar, S. S. (2020). Alexa as Coach: Leveraging Smart Speakers to Build Social Agents that Reduce Public Speaking Anxiety. Conference on Human Factors in Computing Systems - Proceedings, 1–13. https://doi.org/10.1145/3313831.3376561
- Watson, D. R., Garfinkel, S. N., Van Praag, C. G., Willmott, D., Wong, K., Meeten, F., & Critchley, H. D. (2019). Computerized Exposure Therapy for Spider Phobia: Effects of Cardiac Timing and Interoceptive Ability on Subjective and Behavioral Outcomes. Psychosomatic Medicine, 81(1), 90–99. https://doi.org/10.1097/PSY.0000000000000646
- Wrzesien, M., Botella, C., Bretón-López, J., Del Río González, E., Burkhardt, J. M., Alcañiz, M., & Pérez-Ara, M. Á. (2015). Treating small animal phobias using a projective-augmented reality system: A single-case study. Computers in Human Behavior. Elsevier Ltd. https://doi.org/10.1016/j.chb.2015.01.065

14. Transferencia del conocimiento

El software resultante de la implementación del asistente terapéutico conversacional.

Anexar a esta solicitud: Cronograma de actividades

ACTIVIDADES	Año	2019		2020				2021				2022				2023	
	Trimestre	3	4	1	2	3	4	1	2	3	4	1	2	3	4	1	2
**Revisión del estado del arte**		■	■	■	■	■	■	■	■	■	■	■	■	■	■	■	■
**1. Analisis de entendimiento de una TE**		■	■	■	■							■					
Revisar literatura sobre TE		■	■									■					
Tipos de terapias de exposición		■	■									■					
Identificar fases, variables y características			■	■	■							■					
Identificar instrucciones, preguntas y expresiones			■	■	■							■					
**2. Diseño de interacción Paciente-ATIC**					■	■						■					
Identificar y seleccionar las variables del contexto					■	■						■					
Analizar relación entre variables y su objetivo					■	■						■					
Diseñar un ambiente de simulación de una TE					■	■						■					
Evaluar el diseño de interacción Paciente - ATIC					■	■						■					
**3. Prototipo de ATIC**						■	■	■	■			■	■	■	■		
Computó consciente de contexto						■	■					■	■				
Agente conversacional						■	■	■	■			■	■	■			
Aprendizaje autómatico							■	■	■	■			■	■	■		
**4. Evaluación del modelo de ATIC**										■	■				■	■	
Tamaño de muestra										■					■		
Medidas										■					■		
Tratamientos y herramientas										■	■				■		
Procedimiento del TE										■	■				■	■	
Análisis estadísiticos											■					■	
Resultados											■					■	
**Otras actividades**		■	■	■	■	■	■	■	■	■	■	■	■	■	■	■	■
Redacción de tesis				■	■	■	■	■	■	■	■	■	■	■	■	■	■
Presentación de avances			■		■		■		■		■		■		■		■
Publicaciones								■	■						■	■	
Defensa doctoral																	■

Me comprometo a que el Proyecto de Intervención será realizado dando cumplimiento a las normas institucionales y leyes vigentes. Así como, informar oportunamente al Comité de Investigación y Ética de Pregrado y Posgrado, cualquier problema no previsto o de la ocurrencia de eventos adversos serios que impliquen cualquier principio ético.

Dra. María Victoria Meza Kubo
Firma del Director del Proyecto

Sonia Maribel González Lozoya
Firma del Estudiante
