# Supplementary material for: An internet-based self-applied rat phobia treatment using a Virtual Therapy Assistant: Study protocol for a randomized controlled trial
Source: PLoS One. 2023 Feb 15;18(2):e0281338. doi: 10.1371/journal.pone.0281338 (PMC9931099; doi:10.1371/journal.pone.0281338)
Supplement: S2 Protocol — (PDF) [file pone.0281338.s003.pdf]

Registration no. 001/

1. Title of the Intervention Project

Self-applied exposure treatment for rat phobias guided by a Virtual Therapist Assistant

2. Researchers/Identification

RESEARCHERS	No. Emp / Enrollment	Phone(ext.)	Email
María Victoria Meza Kubo	15439	6461173317	mmeza@uabc.edu.mx
Alberto Leopoldo Morán y Solares	13399		alberto.moran@uabc.edu.mx
Cristina Ramírez Hernández			cramirez@ite.edu.mx
Alejandro Domínguez Rodríguez			alejandro.dominguez.r@campusviu.es
Sonia Maribel González Lozoya	00364101	2221170678	sonia.gonzalez68@uabc.edu.mx

3. Participating institutions

Institution (Company name and address)	Subjects of study	Did they give approval to the project?
Instituto Tecnológico de Ensenada<br><br>Universidad Internacional de Valencia España	Adults 18-60 years of age with some level of small animal phobia. That they have a measure of severity of rat phobias between 1 to 3 on a scale of 0 to 4, where 0 is non-phobic, and 4 being the degree of high level of phobia.	Yes<br><br>Yes

4. Funding sources

Institutional	X	Specify: Autonomous University of Baja California
Governmental	X	Specify: CONACYT, Scholarship for doctoral students
Commercial		Specify:
Other		Specify:

5. Abstract(Limit 300 words)

The research work proposes exposure treatment through a virtual therapeutic assistant called VTA (acronym in English: Virtual Therapy Assistant), aware of the context that interacts verbally with the patient, to guide and control different types of exposure therapies for phobias to small animals, while analyzing the physiological records of the patient in real time to determine their emotional state during therapy.

In this study, it is proposed to evaluate case studies where the virtual assistant allows gradually guiding an exposure treatment for rat phobias, taking advantage of intelligent devices for patient monitoring and being considered to determine the progress of treatment.

The proposed therapy is designed based on the literature [Ruiz & Valero, 2017; Flobak et al., 2019; Moran et al., 2019; Bottle et al., 2000; Campos et al., 2019] adapted to integrate VTA, with the goals of increasing the accessibility, motivation and acceptance of ET for rat phobias.

6. Introduction

Specific phobia is a common emotional, cognitive and behavioral mental disorder that affects society. According to the Scientific and Technological Information Agency for the Congress of the Union (INCyTU), it is reported that 7% of the population suffers from a specific phobia, and this begins at an early age of approximately 9 years [Rentería-Rodríguez, 2018]. The specific phobia produces an excessive and irrational fear towards objects or situations in particular, where its physical or mental exposure to the phobic stimulus can cause a panic attack and anxiety manifesting different physical symptoms such as: shortness of breath, sweating, acceleration of heart rate, chest pain or choking sensation, and/or behavior alteration in order to avoid the object or situation that provokes the phobia [Gilbert, 2002].

Among the specific phobias, there are phobias towards small animals: arachnids, insects, rodents and reptiles are the most common. Small animals have been found to become feared for their sudden movements, their physical appearance, the sounds they make, and their tactile properties. Generally, people who suffer from these types of phobias try to anticipate the problem by avoiding exposing themselves, affecting daily activities in different areas: work, academic and social [Bados, 2017].

Currently, one of the most effective treatments to overcome specific phobias is behavioral therapy, where the patient is repeatedly and gradually exposed to their fear. The objective of the therapy is focused on desensitizing the patient, recreating different situations that cause the activation of the reactions of the phobia. This type of therapy is known as Exposure Therapy (ET). The components that constitute an ET are the consensual participation of the patient, the therapist and the presence of the object of fear, where all three are essential for the effectiveness of the therapy.

7. Problem Statement

The problem that exists in using ET for phobias of small animals is that a gradual exposure cannot be ensured, because it is difficult to control the behavior of the feared animal, more so if it is a type of animal that cannot be domesticated, creating the possibility of further aggravating the patient's phobia. This possibility makes ETs for small animal phobias have a low rate of acceptance and a high rate of abandonment [Gilbert, 2002]. Also, there are other types of aspects to consider that mean that between 60% and 80% of people who suffer from a phobia do not seek treatment [Wrzesien et al., 2015], this may be a problem derived from the availability of experts, long-distance transfers, health, economic and social situations [Bados & García, 2011].

Efforts have been made to improve ETs for small animal phobias by incorporating different types of technologies, mainly in symbolic ETs where the patient has contact in a non-real phobic situation or object, using representations or abstractions through different types of elements such as: visual, audio, audiovisual, games and simulations recreating virtual phobic situations or objects [Ruiz et al., 2012]. Symbolic ETs allow the recreation and control of environments (Horváthová et al., 2016; Miloff et al., 2019) and non-real phobic objects (Botella et al., 2011; Botella et al., 2016; Wrzesien et al., 2015) , providing the patient with confidence and security, motivating him to face his fear. Meanwhile, the incorporation of information technologies such as self-help applications through the Internet allows greater accessibility to different psychological treatments, alleviating the aforementioned problems. Examples of application developments that contribute to treatments for specific phobias using means of virtual communication on the Internet are [Donker et al., 2019; Müller et al., 2011; Hnoohom & Nateeraitaiwa, 2017; Andersson et al., 2009]. However, areas of opportunity have been detected that could in turn be seen as desirable features to add to systems that support ETs, which are mentioned below:

A. User centered system
    a. A personalized exposure treatment according to the intensity of severity of the phobia and symptoms manifested by the user.
    b. Analysis of data from the context of the patient that could be useful to determine the appropriate advance of treatment according to the progress achieved [Arquissandas et al., 2019].
B. Self-applied TE through a virtual assistant
    a. A system that allows carrying out an exposure treatment for phobias of small animals through verbal interaction, both oral and textual, and using audiovisual support elements.

In this sense, the proposal of this work is to evaluate a virtual therapeutic assistant that is capable of guiding the patient through a self-applied gradual exposure treatment for small animal phobias, using: context-aware computation and different types of support elements such as: images, videos, interactive games and immersive videos using real scenes through the use of 360º videos, applying sessions remotely. Under this scheme, gradual ET through the use of interactive multimedia with Thera seeks to attract and maintain the user's interest through adaptable sessions and increase the accessibility of self-applied treatments to overcome small animal phobias [Botella et al., 2000; Fields et al., 2019].

8. Intervention project Goals

To evaluate case studies where an intelligent assistant allows to gradually guide an exposure treatment for rat phobias, taking advantage of intelligent devices for monitoring the patient and being considered in the progress of the treatment and in the intensity of the phobic stimulus. The protocol is based on the literature [Ruiz & Valero, 2017; Flobak et al., 2019; Moran et al., 2019; Bottle et al., 2000; Campos et al., 2019] adapted to integrate Thera, with the goals of increasing accessibility, motivation and acceptance of ET for small animal phobias.

9. Frame of reference

Exposure Therapies (ET)

People with specific phobias, such as phobias of small animals, often have fear or disgust caused by traumatic experiences, misinformation, observation of the behavior of others close to them, or just a sense of threat, causing the person with the phobia to avoid a confrontation with the animal. feared animal [Abramowitz et al., 2019]. Avoiding a confrontation with the feared animal is one of the main factors that maintains the phobia and can even cause it to increase in intensity over time. For this reason, exposure therapies are one of the most efficient, since their objective is to expose the patient, in feared situations or objects, through techniques that allow the patient a gradual or intense exposure. The purpose of the TEs is: eliminate negative beliefs or ideas, manage emotional responses, reduce and/or eliminate physiological alterations, disregard the use of safety behaviors and inhibit feared situations.

Types and techniques of exposure therapies

There are different types of exposure therapies that can be used depending on the intensity of the phobia, different contexts and objectives. [Bados and García, 2011] mentions seven types of exposure therapies, which in turn [Ruiz et al., 2012] includes them in two categories of therapies:

1. Live ET, which consists of the patient having real direct contact with a feared situation or object, either gradually or flooding, that is, putting the patient in a situation of high level of anxiety.

2. Symbolic ET where the patient has contact in a feared but not real situation or object, but rather through representations or abstractions. Imagination, audiovisual elements (drawings, photos, videos, recordings), games, simulations of feared situations or substitution of the feared object can be used. Many of these representations are achieved with the help of technology such as virtual reality and augmented reality.

There are several practical techniques that have been developed to overcome phobias and that can be implemented within an ET. The most used techniques are the following [Shunnaq & Raeder, 2016]:

- **Systematic desensitization therapy**: exposure technique with the aim of gradually exposing the patient to their fear, to the phobic stimulus. This technique initially focuses on the patient's acceptance of the treatment. Subsequently, levels of gradual exposure to the object of fear are established according to the

patient, where the lowest level is harmless and the highest level is a representation where a maximum fear response is obtained. And finally, the patient, being calm and relaxed, gradually exposes himself to the object of fear from the lowest level to the highest level established by the therapist. The progress of each level depends on the acceptance of the patient, that is, that he gets used to and supports the previous level in a peaceful way.

- **Flood and implosion therapy**: exposure technique where the patient is presented with the highest level of the object of fear, usually in a real situation. Sometimes it is difficult to define exactly a situation because it can be replaced by images, sounds, videos, virtual reality or others that the therapist considers appropriate. At the same time, when the patient is faced with the level of fear, they are asked to report in detail what they are experiencing in order to find out the origins of the fear and become disinhibited.
- **Interoceptive Therapy**: This is a common technique implemented in combination with other exposure techniques mentioned above. This therapy aims to control the physical symptoms produced by the phobia by implementing breathing techniques or other similar techniques. The technique is intended to reassure and relax the patient before the object of fear.

There are different problems in ET for animal phobias. And it is that despite being one of the most effective treatments, they have a low rate of acceptance and a high rate of abandonment. Because ETs are effective due to gradual exposure to fear objects, however, when confronted with an animal, especially if it is a non-domesticatable animal, gradual exposure cannot be guaranteed and there is the possibility of aggravating the patient's phobia and/or physical injury when fleeing from the fearful situation.

Seeking to alleviate the aforementioned problem, the development of technology has been resorted to that seeks to easily deceive the human senses with the use of immersive technologies, generating applications that serve as support tools in ETs. The advantages of these applications is that they allow you to build a controllable environment and have the ability to recreate and replace the real object of fear. This type of environment gives the patient greater confidence and security to face her fear, increasing the acceptance rate and lowering the abandonment rate in ET.

Some technological applications that support TEs are detailed below:

Computer Based Exposure (CBE)
The use of the computer to carry out an ET for phobias allows exposing the user to different types of elements such as visual, audio, audiovisual content of the phobic stimulus. CBE allows for self-help treatment, provides greater access to treatment, and minimizes the involvement of a therapist.

Here are some jobs that employ computer-based exposure therapy:
(Muller et al., 2011). They conducted a study to assess the effectiveness of a one-session computer-based treatment in reducing fear and avoidance behavior toward spiders by exposing patients to images of spiders compared to neutral images. The study demonstrated compared to a control group, that CBE therapy had a greater reduction in fear before and after treatment, both at the subjective level and at the behavioral level.
(Andersson et al., 2009). They conducted a study comparing an internet-guided self-help treatment with a live session treatment in a sample of patients with spider phobia. The online treatment consisted of five weekly text modules, presented on a web page, a video modeling the presentation, and online support. The results showed that the groups did not differ at post-treatment or follow-up, concluding that Internet-guided exposure treatment is a promising new approach in the treatment of spider phobia.
(Watson et al., 2019). The authors tested the hypothesis that interoceptive information related to cardiovascular arousal may influence outcomes of computerized exposure therapy for spider phobia. The ET consisted of the participants observing four blocks of images of spiders. Each block contained 40 images (35 spiders and 5 abstract shapes). Randomly selected from a bank of 220 spider images, representing a selection of spiders from around the world. Concluding that interoceptive information does influence the benefit of exposure.

Virtual Therapist Assistants

The development of technological tasks such as voice and speech recognition has allowed the creation and use of virtual assistants such as Alexa, Siri, Cortana, among others. These virtual assistants that support us in simple routine tasks minimizing time and effort, have given rise to new proposals for virtual assistants specialized in a field, such as in the area of clinical health. Here are some jobs:

(Wang et al., 2013). They proposed EuTalk™ which is a virtual therapist and speech assistant developed to help people with communication disabilities by supporting communication needs, providing highly accessible rehabilitation regimens.
(Wang et al., 2018). They published another study where they used EuTalk™, concluding that all the participants agreed that the app that included EuTalk™ established an alternative treatment protocol usable for communication rehabilitation.
(Buinhas et al., 2019). Another study with the aim of increasing adherence to medication, physical activity, diet, and adjusting the intervention to the needs of users and their characteristics, used a virtual assistant capable of speaking and expressing emotions through facial and body animations. Validated by academic nurses with experience in primary care, positive opinions and suggestions for improvement were obtained.
Other studies that use virtual assistants in the health sector have shown benefits such as adherence to oral antidiabetics in older people **(Félix et al., 2019)**, or helping patients with dementia and memory loss to identify and recall critical memories **(Tiong, et al., 2018)**, and practice cognitive reconstruction exercises to reduce public speaking anxiety **(Wang, et al., 2020)**.

10. Phase I: Main results of the situational diagnosisshare

Intellectual property

The researchers involved in this study agree to share the intellectual property of the developed application, which will be shared with the Autonomous University of Baja California, which has provided the necessary resources for its development. In addition, the research papers that are submitted to journals and/or congresses will have the researchers involved as co-authors and the order of authorship will be defined with respect to the specific contribution of the published document. In all cases, preference will be given to the main researcher of this doctoral thesis, to appear as first author, as long as her contribution warrants it.

11. Phase II: Planning

Evaluation design

We propose to carry out an evaluation with independent groups, i.e., Randomized Control Trial (RCT). The RCT is considered the "gold standard test" for evaluating the efficacy of interventions (McGovern, 2006). The RCT is a type of study where participants are randomly assigned to one of two or more groups of clinical interventions. Each group will participate in one of the following conditions (Each group will consist of 10 subjects):

- Group 1: Control group or waiting list, participants in this group will not receive treatment immediately. The measurements of the participants will be obtained for the first time and after a month a second time. With the purpose of comparing if there were changes when not receiving any intervention.
- Group 2. Intervention group with self-applied exposure treatment for rat phobias guided by Thera. The participants in this group will have a maximum of one month to carry out the treatment, which must overcome four stages of gradual exposure, taking measurements before and after the treatment for comparison.

The evaluation will consist of the following phases:

1. Recruitment and selection of participants

The participants in this study will be adults according to Mexican law and who volunteer to participate in it. The study will be published in different media such as social networks (facebook, whatsapp and twitter) and the media of the institutions of the researchers participating in this study for its dissemination and to reach more potential participants. Potential participants may contact the study recruiters by email, whatsapp or phone call. Any Mexican person who understands Spanish can benefit from the intervention, as long as they meet the following inclusion and exclusion criteria.

The criteria considered for the selection of participants are the following:

- Inclusion criteria
  - Men and women from 18 to 60 years old.
  - Meet the diagnostic criteria for specific phobia towards rats (with a mild and moderate degree), obtained by the structure of interviews for anxiety disorders, in the English Anxiety Disorder Interview Schedule (ADIS) of DSM-IV [Brown and Barlow, 2014].
  - Have basic digital knowledge and skills (computer and internet management).
  - Have the basic technological devices: email, computer/laptop, smartphone, microphone, internet connection and bluetooth.
- Exclusion criteria
  - Receive another type of psychological or psychopharmacological treatment.
  - Being diagnosed with another type of anxiety disorder or some psychopathology.
  - Have a medical condition that puts your life at risk (heart disease, respiratory disease, pregnancy, among others).

To verify that these criteria are met, an ADIS-IV structured interview will be applied by videoconference [Brown et al., 1994].

2. Prior to intervention

a. A signed informed consent form was requested from each of the participants. The content of the format consists of informing the participant of the instructions, place, time and approximate duration of the intervention. Also, inform the risks and concerns involved, benefits, information that will be recorded and the protection of said information that you share with us during the intervention. With the signed consent form of the participant, the information collected during the study will generate a personal file. All files will be stored according to ethical conditions with full support and only the main researcher will have access to the full name, while the associated researchers will have access to the results obtained but not to identification data. The files will be kept in complete protection for a minimum period of five years, it is clarified that the databases with the scores obtained in the instruments do not have identification data. And all information presented in discussion forums will maintain global average results, but not individual scores or personal identification data.

b. The participant is contacted and invited to go to the specific place to attend the intervention where the necessary devices will be available to implement the treatment. The devices are: (1) SmartWatch, used especially for real-time heart rate acquisition of the participant; (2) VTA application, which allows a verbal interaction of the participant to determine the progress of the ET session; (3) device (PC, laptop, tablet) with: VTA application and eye detection model, internet connection, camera and microphone to observe and listen to the participant's behavior and bluetooth connection to establish communication between the devices; and, (4) Smartphone and Google Cardboard for 360º video immersion.
c. The participant is instructed about the intervention space: stand in front of the device and access the VTA application where the participant can listen and respond, put on the SmartWatch and synchronize it to obtain the heart rate.
d. The baseline of the participant is established, obtaining the previous measurements: fear and avoidance of rats, heart rate reading and eye detection.

3. Intervention

A. *VTA located in the local computer equipment, starts by synchronizing the communication between the SmartWatch, camera and microphone devices. In the event that the synchronization fails or there is any other technical problem, VTA must provide information for technical support and/or instructions on how to solve the problem.*

B. Subsequently, VTA creates the participant's record that, by filling out forms, allows knowing how familiar the participant is with interacting with a virtual assistant and obtaining general user data and basic information such as:

a. Level of fear of rats through the questionnaire (FRQ: acronym in English "Questionnaire on the fear of rats"). Questionnaire adapted from (FSQ; Fear of Spider Questionnaire; Szymanski & O'Donohue, 1995).
b. Level of anxiety in rats through the Trait-State Anxiety Inventory (IDARE; Díaz-Guerrero & Spielberger, 1975).
c. Other fears through the Fear Questionnaire (FQ; Fear Questionnaire; Marks & Mathews, 1979).

In the event that the participant is already registered, information is obtained on her progress such as the number of sessions, physiological reactions during her exposure, progress and progress of the therapy. With the value of the severity of the phobia, the level of exposure of the phobic object that the participant will initiate is determined.

C. In the case that the participant is not familiar with a virtual assistant, a training stage will be provided to show how it works and what it can do, providing all the possible instructions that can be requested from VTA. With the purpose that the participant feels the confidence of being heard and that he has control of the progress of the program and be assisted at any time. Therefore, it is required that the participant is determined to face her phobias in order to improve her quality of life, reducing the discomfort caused by it.

D. Starting the session, VTA welcomes the participant and informs them about the content and goal of exposure treatment for rat phobias.

E. *VTA classifies the heart rate through the reading of the pulses per minute (ppm) provided by the SmartWatch, to evaluate and determine four different levels: **1) Normal**: the heart rate is within the established normal range and indicates the continuity of the session; **2) Low altered**: heart rate between threshold A and threshold B. Indicates the presence of a mild physiological alteration where the participant can still tolerate the situation and get used to it. **3) Altered high**: heart rate between threshold B and maximum heart rate. Indicates a high physiological disturbance, the exposure therapy is stopped for a moment and VTA proposes to start performing a deep breathing exercise as a relaxation strategy to calm the participant and change his state to low altered or normal; finally, **4) Crisis**: the heart rate exceeds the maximum range. VTA tries to reduce cardiac activity through deep breathing exercise and there is a possibility that the participant decides to end the session for the day. Tables 1 and 2 show the variables used and the ranges established to determine the different categories of the participant's status [American Heart Association, 2020].*

Table 1. Variables considered in heart rate.

Variables	Value
Age	Edad del usuario
Heart Rate Maximum (HRM)	*HRM*= 220 - Age
ThresholdA	A= (*HRM* * 0.50) bpm
ThresholdB	*B*= (*HRM* * 0.85) bpm

bpm: beats per minute

Table 2. User heart rate status classification.

States	Heart rate
**Crisis**	Greater HRM
**altered high**	Between *B* at *HRM*
**altered low**	Between*A* to *B* and minor of 60 or 40 bpm
**Normal**	If you exercise: between 40 bpmat A

	Does not exercise: between 60 bpmat A

F. VTA asks the user if they are ready to start the virtual exposure therapy of the phobic object. If VTA classifies the user's heart rate in a state of high alteration, it will start a deep breathing exercise until it has calmed down, that is, it is at a normal level. If you get a negative answer or do not receive an answer to the question when no alteration is detected, it will wait until the participant has prepared himself by playing relaxing music in the background. If neither of the two previous conditions exist, the established session will continue.

G. In this part of the session the participant is exposed to the phobic object. The exposure is divided into four stages considering a gradual exposure based on the realism, interaction and intensity of the object according to the taxonomy proposed by Morán and collaborators [Morán et al., 2020].

- In the first and second stages, the participant is exposed to the phobic object supported by multimedia material based on the Multimedia Behavioral Avoidance Test (MBAT) instrument. The MBAT is a reactive test where the participant is presented with various visual stimuli (images and videos) related to the object of fear [Ruiz & Valero, 2017].
- In the third stage, the participant virtually interacts with representations of rats, using various levels of gradual exposure in a video game [Hnoohom & Nateeraitaiwa, 2017; Shunnaq & Raeder, 2016].
- Finally, in the fourth stage, the participant, through 360º videos, using a smartphone and google cardboard (accessible elements), is immersed in a virtual reality environment simulating a natural situation (as an example, see the Covid feel good web page, 2020), in where the content of the video will show rats with different types of natural behavior.
-

The stages of the treatment will be scalable, that is, the participant must pass a stage to advance to the next. However, VTA can unlock some of the early stages by comparing the participant's severity rating with the severity rating established at each stage of treatment.

The content of each stage was fed back and validated by specialists in intervention to phobias. Each stage is described in detail below.

a. ***First stage: gradual exposure through images***

In the event that the participant has a phobia of moderate severity, it is recommended that the exposure begin visually through images. The purpose of this stage is for the participant to get used to and tolerate the appearance of the rats, through 10 images where each of the rats is observed for at least 10 seconds, a procedure adapted from Ruiz & Valero [2020].

The treatment process in the first stage is detailed below:

a. VTA informs the participant of the content and objective of the first stage.
b. VTA informs the participant that it will show him an image, describe the content and instruct him that he needs to look at it for 10 seconds.
c. VTA asks the participant if he is ready to view the image.
d. In case the participant answers negatively, VTA tells him that it will give him 1 minute to get ready and will ask again. Otherwise, VTA will show you the corresponding image.
e. VTA classifies the heart rate of the participant (process explained above in point 8).
f. At the end of 10 seconds, if the participant did not show an alteration in their heart rate and looked at the screen without hesitation, VTA will continue with the next image. Otherwise, VTA will inform you that it will show you the same image again until you get used to it and bear to look at the image.
g. Steps b to f are repeated until the participant can observe the ten images described in Table 3 without getting upset.
h. In the middle (after the fifth image) and at the end of the first stage, VTA will ask the participant to answer the following questions with the greatest sincerity: using a scale from 1 to 10 where 1 is nothing and 10 is

a lot, how much Do you think you were able to observe the rats in the images? and what is the intensity of anxiety that he considers that he has experienced with the images shown?

i.

Table 3. Content of the images that will be shown in stage 1.

	Realism	Intensity			Visual interaction
Image	Kind of image	Number of rats	Size	Appearance	Observe content
1	Caricature drawing	1	Small	kind	a single rat
2	Realistic drawing	1	Median	inoffensive	a single rat
3	Photo	1	pequeña	inoffensive	a single rat
4	Photo	1	Median	inoffensive	a rat on a person's hand
5	Photo	1	Median	inofinoffensi veensiva	a single ra
6	Photo	1	Big	inoffensive	a rat on a person's shoulder
7	Photo	1	Big	not nice	hungry rat
8	Photo	3	Big	not nice	rat with pups
9	Photo	more than 4	Big	not nice	rats in the trash
10	Photo	many	Big	not nice	rat colony

b. Second stage: gradual exposure through videos

In the event that the participant has a phobia of moderate to mild severity, it is advisable to make an audiovisual presentation through videos. The purpose of this stage is that the participant can get used to and tolerate the natural appearance and behavior of the rats through the observation of 6 short videos with a duration of 1 minute according to the video, a procedure adapted from Ruiz and Valero [2020].

The treatment process in the second stage is detailed below:

a. VTA informs the participant of the content and objective of the second stage.
b. VTA informs the participant that it will show a video, describes the content and its duration, and that it is necessary to watch it at all times.
c. VTA asks the participant if he is ready to watch the video.
d. In case the participant answers negatively, VTA tells him that it will give him 1 minute to get ready and will ask again. Otherwise, VTA will show you the corresponding video.
e. VTA assesses the emotional state of the participant (process explained above in point 8).
f. If the participant did not show an alteration in their heart rate and watched the screen for the duration of the video transmission, VTA will continue with the next video. Otherwise, VTA will inform you that it will show you the same video again, until you get used to it and can bear to watch the entire video.
g. Steps b through f are repeated until the participant can watch the six short videos described in Table 4 without being disturbed.
h. In the middle (after the third video) and at the end of the second stage, VTA will ask the participant to answer the following questions with the greatest sincerity: using a scale from 1 to 10 where 1 is nothing and 10 is a

lot, how much do you consider What did the rats observe? And what is the intensity of anxiety that he considers that he has experienced with the videos shown?

i.

Table 4. Content of the videos that will be shown in stage 2.

	Intensity	Interaction	Realism
Video	Number	Watch video content	Aspect
1	1	How to draw a rat	drawing
2	1	friendly rat	cartoon childish
3	1	Rat and his cage	real
4	More ofe 1	Rat with its young and its keeper	real
5	More of 1	rat community	realistic caricature
6	More of 1	street rat community	real

c. Third stage: Gradual interaction with virtual rats.

In case the participant has a mild severity phobia of rats, gradual exposure can be achieved through a serious game using virtual rats. The purpose of the video game stage is that the participant can approach, observe and interact with different virtual rats through a first-person video game, where the participant's movements are directed through the PC keyboard. As an example, Figure 1 shows a virtual scene, where the participant is interacting with rats in the first person. Some related works have shown that games are effective in treating other types of specific phobias, such as dogs or spiders [Hnoohom & Nateeraitaiwa, 2017; Shunnaq & Raeder, 2016].

The video game consists of five levels that increase the intensity of exposure through the appearance and size of the virtual rat, as shown in Table 5. The game 's setting is in a house that has recently been acquired, where the participant must explore and find rats that you will have to get rid of to inhabit your new house. During the exploration of the house, the participant will find different rats that will activate mini-games with 3D rat models to acquire items that will later help to capture them. Overcoming the minigames will increase the size and intensity of speed and movements of the rats. The participant, by capturing a certain number of rats, will be rewarded by improving and unlocking other spaces in his house.

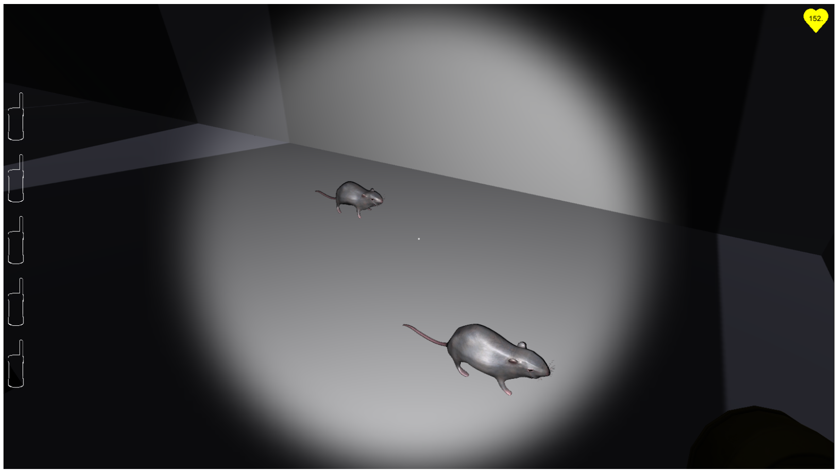

Figure 1. Example of virtual interaction with rats, with first person impression.

Table 5. Aspect and size of the virtual rats according to the level of exposure of the video game.

Nivel	Aspect	Size

1	animated	Small
2	animated	Median
3	real	Small
4	real	Median
5	real	Big

The treatment process in the third stage is detailed below:

a. VTA informs the participant that the main objective of the stage is to be able to support and face feared situations with exposed rats through a video game. Later, it informs you about the context and instructions necessary to be able to play the game, for example how to move and pick up objects in the game. Also, it will explain that on the left side of the screen items that you find will be activated and on the upper right side of the screen, a heart will be shown whose color will change according to your emotional state: green, if you are calm or normal; yellow, if it is altered low; orange, if it is high altered; and red, if you are in crisis.
b. If VTA detects the normal participant or only a little upset (low upset), VTA asks if they want to start the game.
c. If the participant's answer is positive, VTA starts the game, but if the answer is negative or no answer was obtained, VTA will wait 15 seconds to ask the question again. In the event that VTA has asked three times and does not obtain a positive answer, VTA will ask if they wish to continue with the session, to which if the participant does not answer positively, VTA will end the session.
d. The game begins and the participant appears at the front door of the house.
e. VTA instructs the participant that the only way to inhabit their new house is to get rid of the rats, and for this it is necessary to gather courage and explore the spaces of the house.
f. When the participant is touring the unlocked spaces of the house, they will meet different rats that as the participant finds and captures the rats, the rat exposure will increase the movements, displacement, quantity and frequency of appearance. Table 6 shows the gradual behavior and interaction of the rats according to the number of rats captured by the participant.
g. In the middle (after the third level) and at the end of the game, VTA will ask the participant to answer the following questions with the greatest sincerity: using a scale from 1 to 10 where 1 is nothing and 10 is a lot, how much do you consider were you able to observe the 3D rats? And what is the intensity of anxiety that he considers that he has experienced in the video game?

Table 6. Behavior and gradual interaction of virtual rats in the video game.

**Nivel**	**Gradual behavior and interaction of virtual rats**
1	The participant has not captured any rats, the frequency and speed of the rats within the minigame will be slow to the size of animated looking girls.
2	The participant has captured 1 to 3, the frequency and speed of the rats within the minigame will be medium slow with medium size of animated aspect.
3	The participant has captured 4 to 6, the frequency and speed of the rats within the minigame will be moderate with life-size rats.
4	The participant has captured 7 to 9, the frequency and speed of the rats within the minigame will be moderate with medium size of real appearance.
5	The participant has captured 10 to 11, the frequency and speed of the rats within the minigame will be fast with large lifelike size.

h. At the moment the participant captures a rat, VTA congratulates him, checks the duration of the minigame, if the duration was less than 1 minute and he did not have any physiological alteration, he gives him a new item for home. Afterwards, VTA informs the participant to continue exploring and. In the event that the participant has captured the last rat, a short video will be shown where the participant can observe all the spaces in his house cleaned, remodeled with all the items won.

d. Fourth stage: Immersion in a Virtual Reality (VR) environment.

In case the participant has a mild severity phobia of rats, they can make a VR exposure by using glasses and a 360º video. Using different videos of urban or rural scenes, where rats are observed around. The purpose of this stage is that the participant can immerse himself and be able to bear observing the behavior of the street rats that are around the scene, as an example [Flobak et al., 2019] who created prototypes of virtual reality scenarios for TE, enabled by 360º video based on lived experiences.

The treatment process in the fourth stage is detailed below:

a. VTA informs the participant of the content and objective of the fourth stage.
b. VTA informs the participant that it will show them a 360º video using a Google cardboard that allows a VR immersion, describes the content of the video and instructs them that it is necessary to observe in detail according to the duration of the video.
c. VTA assesses the emotional state of the participant (process explained above in point 8).
d. If the participant did not show an alteration in their heart rate and watched the scene during the duration of the video transmission, VTA will continue with the next video. Otherwise, VTA will inform you that it will show you the same video again, until you get used to it and bear watching the video.
e. Steps b, c, and d are repeated until the participant can watch six short videos described in Table 7 without getting upset.
f. After observing each immersive video, VTA will ask the participant to answer the following questions as honestly as possible: using a scale from 1 to 10 where 1 is not at all and 10 is a lot, how much do you think you were able to observe the rats? and what is the intensity of anxiety that he considers that he has experienced the immersive video?

Table 7. Content of the 360º videos that will be shown in stage 4.

Video	Duration	Scene	Observe content
1	3 min	Urban, in crowded places in the city	Showing one or more real rats in the distance
2	3 min	Inside the home	A real rat appears walking in the room

I. At the end of each of the treatment stages, VTA will analyze various aspects to determine if the participant has passed the corresponding stage. Checking the following:

1. Physiological alteration: during the corresponding stage the participant has not had a high alteration or that most of the stage will be at a low altered level.
2. Avoidance: a) the time that the stage lasted, b) the way of answering, either positively or negatively to show the following exposure elements (images, videos, video game levels or immersive videos), and c) subjective avoidance unit, provided by the participant in the middle and at the end of each stage.
3. Subjective unit of anxiety, provided by the participant in the middle and at the end of each stage.

If VTA determines that the participant passed the stage, he will start the next one or will have finished the TE if the stage passed was the last one, but if VTA determines that the participant did not pass the stage, he will repeat it again.

4. Evaluation

Once the treatment for rat phobias has finished or the maximum period of four days has elapsed, the participants will be asked to complete the following surveys:

a. *Questionnaire on the fear of rats (FRQ): a questionnaire adapted from the Fear of Spider Questionnaire (FSQ; Szymanski & O'Donohue, 1995).*

b. *State-Trait Anxiety Inventory (IDARE): The Spanish version of the STAI (State-Trait Anxiety Inventory) consists of 40 statements; 20 evaluate trait anxiety and 20, state anxiety. It has been validated in Mexico by Rojas-Carrasco (2010), obtaining adequate reliability for the two dimensions of anxiety (Cronbach's alpha coefficients greater than 0.83).*

c. *Fear Questionnaire (FQ; Fear Questionnaire; Marks & Mathews, 1979): evaluates the intensity with which certain situations are avoided and worried, and also contains a scale where the patient assesses the current state of their phobic symptoms.*

d. *Sense of presence and judgment of reality: this instrument evaluates the degree of presence experienced through the visual elements through two questions with a Likert scale from 0 to 10 (0 = not at all, 10 = too much) (Juan et al., 2005) : 1) To what extent have you felt present in the immersion? and 2) To what extent have you felt in a place where rats appeared?*

e. *Client Satisfaction Questionnaire (CSQ-8S): consists of 8 questions with a total score ranging between 8 and 32 to measure the level of satisfaction with the treatment and has been used to evaluate self-applied treatments (Palma-Gomez , et al., 2020). The question "To what extent did you find him aversive?" was added to this questionnaire (Botella et al., 2009).*

f. *Telehealth Usability Questionnaire (TUQ): evaluates the usability of telehealth systems (Parmato et al. 2016), through two dimensions: 1) effectiveness and 2) ease of use. It contains 12 questions with a Likert 7 scale ranging from 1 to 7 (1 = disagree, 7 = agree).*

g. *Patient Improvement Scale CGI-I (Global Clinical Impression Scale, CGI; Guy, 1976): contains an item adapted from the CGI scale and allows the patient to assess their degree of improvement on a 7-point scale (1 = much better , 7 = much worse).*

h.

12. Protection of participant information

Participants will be assessed individually and only personnel trained in the application of instruments will have direct contact with the participant. They will be informed before proceeding with any application about the use of the data, the freedom they have to abandon the study if they wish to do so at any time during the process, and finally they will decide whether or not they wish to participate in the protocol, giving their authorization or rejection in writing to the evaluator.

A personal file will be made of those who participate in the application of the tests, only in the consent will their name and signature be specified and in the rest of the document their initials will be handled. All files will be stored in accordance with ethical conditions with full support and only the main investigator will have access to the full name, while the associated researchers will have access to the results obtained but not to identification data. The files will be kept in complete protection for a minimum period of five years, it is clarified that the databases with the scores obtained in the instruments do not have identification data. And all the information presented in discussion forums will maintain global average results, but not individual scores or personal identification data.

The files generated in digital format will be filed in a folder with access permission only to authorized personnel granted by the principal investigator. Said files will be protected for at least 5 years for later destruction.

13. Bibliographic references

- Abramowitz JS, Deacon BJ, Whiteside SPH. (2019). Exposure Therapy for Anxiety: Principles and Practice. Second Edi. New York: The Guilford Press. www.guilford.com.
- Andersson, G., Waara, J., Jonsson, U., Malmaeus, F., Carlbring, P., & Öst, L. G. (2009). Internet-based self-help versus one-session exposure in the treatment of spider phobia: A randomized controlled trial. Cognitive Behavior Therapy, 38(2), 114–120. https://doi.org/10.1080/16506070902931326
- Arquissandas, P., Lamas, D., & Oliveira, J. (2019). Augmented Reality and Sensory Technology for Treatment of Anxiety Disorders. *2019 14th Iberian Conference on Information Systems and Technologies (CISTI)*, (June), 1–4. https://doi.org/10.23919/cisti.2019.8760859
- Bados, A. (2017). Fobias Específicas. Barcelona, España.
- Botella, C., Quero, S., Serrano, B., Baños, R. M., & García-Palacios, A. (2009). Avances en los tratamientos psicológicos: La utilización de las nuevas tecnologías de la información y la comunicación [Advances in psychological treatments: The use of new information and communication technologies]. Anuario de Psicología, 40(2), 155–170.
- Botella, C., Breton-López, J., Quero, S., Baños, R. M., García-Palacios, A., Zaragoza, I., & Alcaniz, M. (2011). Treating cockroach phobia using a serious game on a mobile phone and augmented reality exposure: A single case study. *Computers in Human Behavior*, *27*(1), 217–227. https://doi.org/10.1016/j.chb.2010.07.043
- Botella, C., Fernández-Álvarez, J., Guillén, V., García-Palacios, A., & Baños, R. (2017). Recent Progress in Virtual Reality Exposure Therapy for Phobias: A Systematic Review. *Current Psychiatry Reports*, *19*(7). https://doi.org/10.1007/s11920-017-0788-4
- Botella, C., Pérez-Ara, M. Á., Bretón-López, J., Quero, S., García-Palacios, A., & Baños, R. M. (2016). In Vivo versus augmented reality exposure in the treatment of small animal phobia: A randomized controlled trial. *PLoS ONE*, *11*(2), 1–22. https://doi.org/10.1371/journal.pone.0148237
- Botella, C., Baños, R., Guillén, V., Perpiña, C., Alcañiz, M., Pons, A. (2000). Telepsychology: Public Speaking Fear Treatment on the Internet. CyberPsychology & Behavior, 3(6), 959–968. doi:10.1089/109493100452228
- Brown, T. A., & Barlow, D. H. (2014). Anxiety and related disorders interview schedule for DSM-5 (ADIS-5L) : client interview schedule, 114.
- Buinhas, S., Cláudio, A. P., Carmo, M. B., Balsa, J., Cavaco, A., Mendes, A., ... Guerreiro, M. P. (2019). Virtual assistant to improve self-care of older people with type 2 diabetes: First prototype doi:10.1007/978-3-030-16028-9_21
- Campos, D., Bretón-López, J., Botella, C., Mira, A., Castilla, D., Mor, S., Baños, R. & Quero, S. (2019). Efficacy of an internet-based exposure treatment for flying phobia (NO-FEAR Airlines) with and without therapist guidance: a randomized controlled trial. BMC Psychiatry, 19(1), 86–. doi:10.1186/s12888-019-2060-4
- Díaz-Guerrero, R., & Spielberger, C.D. (1975). STAI: Anxiety Inventory: Trait-State. [IDARE: Inventario de ansiedad: rasgo-estado]. El Manual Moderno.
- Donker, T., Cornelisz, I., van Klaveren, C., van Straten, A., Carlbring, P., Cuijpers, P., & van Gelder, J. L. (2019). Effectiveness of Self-guided App-Based Virtual Reality Cognitive Behavior Therapy for Acrophobia: A Randomized Clinical Trial. JAMA psychiatry, 76(7), 682–690. https://doi.org/10.1001/jamapsychiatry.2019.0219
- Félix, I. B., Guerreiro, M. P., Cavaco, A., Cláudio, A. P., Mendes, A., Balsa, J., Carmo, M. B., Pimenta, N., & Henriques, A. (2019). Development of a complex intervention to improve adherence to antidiabetic medication in older people using an anthropomorphic virtual assistant software. Frontiers in Pharmacology, 10, 680 https://doi.org/10.3389/fphar.2019.00680
- Flobak, E., Wake, J. D., Vindenes, J., Kahlon, S., Nordgreen, T., & Guribye, F. (2019). Participatory design of VR scenarios for exposure therapy. Conference on Human Factors in Computing Systems - Proceedings, 1–12. https://doi.org/10.1145/3290605.3300799
- Gilbert, S. (2002). *Coping with Anxiety and Phobias*. Boston. Retrieved from www.health.harvard.edu
- Guy, W. (1976). ECDEU Assessment Manual for Psychopharmacology. Rockville, MD: US Department of Health, Education, and Welfare Public Health Service Alcohol, Drug Abuse, and Mental Health Administration.
- Hnoohom, N., & Nateeraitaiwa, S. (2017). Virtual reality-based smartphone application for animal exposure. 2nd Joint International Conference on Digital Arts, Media and Technology 2017: Digital Economy for Sustainable Growth, ICDAMT 2017, 417–422. https://doi.org/10.1109/ICDAMT.2017.7905004

- Horváthová, D., Siládi, V., & Lackova, E. (2016). Phobia treatment with the help of virtual reality. *2015 IEEE 13th International Scientific Conference on Informatics, INFORMATICS 2015 - Proceedings*, 114–119. https://doi.org/10.1109/Informatics.2015.7377818
- Juan, M. C., Alcañiz, M., Monserrat, C., Botella, C., Baños, R. M., & Guerrero, B. (2005). Using augmented reality to treat phobias. IEEE Computer Graphics and Applications, 25(6), 31–37. https://doi.org/10.1109/MCG.2005.143
- Marks, I. M., & Mathews, A. M. (1979). Brief standard self-rating for phobic patients. Behavior research and therapy, 17(3), 263–267. https://doi.org/10.1016/0005-7967(79)90041-x
- Miloff, A., Lindner, P., Dafgård, P., Deak, S., Garke, M., Hamilton, W., Carlbring, P. (2019). Automated virtual reality exposure therapy for spider phobia vs. in-vivo one-session treatment: A randomized non-inferiority trial. *Behavior Research and Therapy*, *118* (June 2018), 130–140. https://doi.org/10.1016/j.brat.2019.04.004
- Morán, A., Ramírez-Fernández C., Meza-Kubo V. (2020). Design of Smart Objects of Fear with a Taxonomy of Factors Affecting the User Experience of Exposure Therapy Systems for Small-Animal Phobias.
- Müller, B. H., Kull, S., Wilhelm, F. H., & Michael, T. (2011). One-session computer-based exposure treatment for spider-fearful individuals - Efficacy of a minimal self-help intervention in a randomized controlled trial. Journal of Behavior Therapy and Experimental Psychiatry, 42(2), 179–184. https://doi.org/10.1016/j.jbtep.2010.12.001
- Palma-Gómez, A., Herrero, R., Banõs, R., Garciá-Palacios, A., Castañeiras, C., Fernandez, G. L, Llull, D. M., Torres, L. C., Barranco, L. A., Cárdenas-Gómez, L., & Botella, C. (2020). Efficacy of a self-applied online program to promote resilience and coping skills in university students in four Spanish-speaking countries: Study protocol for a randomized controlled trial. BMC Psychiatry, 20(1), 1–16. https://doi.org/10.1186/s12888-020-02536-w
- Parmanto, B., Lewis, Jr., A. N., Graham, K. M., & Bertolet, M. H. (2016). Development of the Telehealth Usability Questionnaire (TUQ). International Journal of Telerehabilitation, 8(1), 3–10. https://doi.org/10.5195/ijt.2016.6196
- Rentería Rodríguez, M. E. (2018). *Salud mental en México. NOTA-INCyTU [Internet] 2018 [acceso 22 de enero de 2019]; 007:1-6* (Vol. 52). Ciudad de México. Retrieved from https://www.foroconsultivo.org.mx
- Rojas-Carrasco, K. E. (2010). Validación del Inventario de Ansiedad Rasgo-Estado en padres con un hijo en terapia intensiva. [The state-trait anxiety inventory for parents who have hospitalized children in an intensive care unit] Revista Médica Del Instituto Mexicano Del Seguro Social, 48(5), 491-496.
- Abramowitz, J. S., Deacon, B. J., & Whiteside, S. P. H. (2019). Exposure Therapy for Anxiety: Principles and Practice (Second Edi). New York: The Guilford Press. Retrieved from www.guilford.com
- Ruiz, A., & Valero, L. (2017). Eficacia de un tratamiento de exposición progresiva multimedia para fobias específicas: un estudio clínico y experimental [Efficacy of a treatment of multimedia progressive exposure for specific phobias: An experimental and clinical study] [Doctoral Dissertation, University of Malaga]. https://hdl.handle.net/10630/15534
- Ruiz-García, A., & Valero-Aguayo, L. (2020). Multimedia intervention for specific phobias: A clinical and experimental study. Psicothema, 32(3), 298–306. https://doi.org/10.7334/psicothema2020.87
- Szymanski, J., & O'Donohue, W. (1995). Fear of Spiders Questionnaire. Journal of behavior therapy and experimental psychiatry, 26(1), 31–34. https://doi.org/10.1016/0005-7916(94)00072-t
- Shunnaq, S., & Raeder, M. (2016). VirtualPhobia: A Model for Virtual Therapy of Phobias. *Proceedings - 18th Symposium on Virtual and Augmented Reality, SVR 2016*, 59–63. https://doi.org/10.1109/SVR.2016.20
- Tiong, E. Y. C., Powers, D. M. W., & Maeder, A. J. (2018). Dementia virtual assistant as trainer and therapist: Identifying significant memories and interventions of dementia patients. ACM International Conference Proceeding Series, 1–9. https://doi.org/10.1145/3167918.3167953
- Wang EH, Chen SH, Zhou L, Hill K, (2013) "EuTalk: A Virtual Therapist and Speech Assistant for People with Communication Disabilities", SDA Conference, University of Pittsburgh, Pittsburgh, PA. (October 31, 2013 - November 1, 2013)
- Wang, E. -., Zhou, L., Chen, S. -. K., Hill, K., & Parmanto, B. (2018). Development and evaluation of a mobile AAC: A virtual therapist and speech assistant for people with communication disabilities. Disability and Rehabilitation: Assistive Technology, 13(8), 731-739. doi:10.1080/17483107.2017.1369592
- Wang, J., Yang, H., Shao, R., Abdullah, S., & Sundar, S. S. (2020). Alexa as Coach: Leveraging Smart Speakers to Build Social Agents that Reduce Public Speaking Anxiety. Conference on Human Factors in Computing Systems - Proceedings, 1–13. https://doi.org/10.1145/3313831.3376561

- Watson, D. R., Garfinkel, S. N., Van Praag, C. G., Willmott, D., Wong, K., Meeten, F., & Critchley, H. D. (2019). Computerized Exposure Therapy for Spider Phobia: Effects of Cardiac Timing and Interoceptive Ability on Subjective and Behavioral Outcomes. Psychosomatic Medicine, 81(1), 90–99. https://doi.org/10.1097/PSY.0000000000000646
- Wrzesien, M., Botella, C., Bretón-López, J., Del Río González, E., Burkhardt, J. M., Alcañiz, M., & Pérez-Ara, M. Á. (2015). Treating small animal phobias using a projective-augmented reality system: A single-case study. Computers in Human Behavior. Elsevier Ltd. https://doi.org/10.1016/j.chb.2015.01.065

14. Transferencia del conocimiento

El software resultante de la implementación del asistente terapéutico conversacional.

I promise that the Intervention Project will be carried out in compliance with current institutional regulations and laws. As well as, promptly inform the Undergraduate and Postgraduate Research and Ethics Committee of any unforeseen problem or the occurrence of serious adverse events that imply any ethical principle.
