# Supplementary material for: An internet-based self-applied rat phobia treatment using a Virtual Therapy Assistant: Study protocol for a randomized controlled trial
Source: PLoS One. 2023 Feb 15;18(2):e0281338. doi: 10.1371/journal.pone.0281338 (PMC9931099; doi:10.1371/journal.pone.0281338)
Supplement: S1 Appendix — (PDF) [file pone.0281338.s004.pdf]

INFORMED CONSENT FORM

To participate in a research project:

Self-applied exposure treatment for rat phobias guided by VTA

Instructions:

"We inform you that you have been invited to participate in the UABC project, which will last approximately 1 hour for 4 days (the duration is subject to change depending on the severity of the phobia and the progress of each participant). For this, it is necessary to carry out a self-applied exposure treatment to the rats with the support of technology, in addition to answering some questionnaires that will be applied before and after the intervention. All information you share with us, as well as registered material, will be used to generate reports and publications of the Project and at no time will confidential or personal information that leads to your identification be used publicly; All reports will be treated completely anonymously. On the other hand, the information you provide us will be very useful and will allow a postgraduate student to graduate. Finally, the protocol that we have designed for this project complies with national (General Health Law), international and regional laws since it has been approved by the Undergraduate and Postgraduate Research and Ethics Committee of the Faculty of Engineering and Business, Guadalupe Victoria".

Procedure:

- If you are willing to participate in the study, we will ask you to sign the following form indicating that you are interested in participating.
- The confidentiality of the information you provide us will be taken care of at all times. These activities will be carried out by an associated student and supervised by the researchers indicated in the confidentiality agreement at the end of this document.
- You can carry out the activity at a time and day that does not interfere with your main activities.
- You are free to withdraw from the evaluation at any time.
- The activities carried out during this study do not contain religious, political or ideological information.

Benefits:
You will benefit from a free intervention that aims to reduce your symptoms manifested by the phobia, thus improving your quality of life. Additionally, you will be notified when, according to the observations, a more extensive assessment process or some other specialized medical or psychological care service is required.

Risks and concerns:
There are no serious risks related to your participation in the study. If you feel unwell or do not wish to continue with the activity, you can withdraw at the time you decide.
If you have any questions about the rights, benefits and responsibilities that you acquire by participating in this study; please contact one of those responsible for the investigation listed at the end of the document.

I,
(participant's name) ____

I agree to voluntarily participate in the project conducted by the UABC researchers listed at the end of this document and I declare that I am of legal age according to Mexican law.

Participante

____
FIRMA

CONFIDENTIALITY COMMITMENT

The researchers of the study for which you have expressed your desire to participate, having given your informed consent, undertake to keep the maximum confidentiality of the information, as well as assure you that the findings and all audiovisual material generated will be used only for research purposes and will not harm them at all.

Sincerely,

____

Dra. María Victoria Meza Kubo
Project Manager
Teléfono: 6461173317
Correo-e: mmeza@uabc.edu.mx
UABC

Co-Responsible

Dr. Alberto Leopoldo Morán
Teléfono: 6461196692
Correo-e: alberto.moran@uabc.edu.mx
UABC

Dra. Cristina Ramírez Fernández
Teléfono: 646108 4095
Correo-e: ramirez@ite.edu.mx
ITE

Dr. Alejandro Domínguez Rodríguez
Correo-e: alejandro.dominguez.r@campusviu.es
VIU, Spain

Associate students

Sonia Maribel González Lozoya
Teléfono: 222 1170678
Correo-e: sonia.gonzalez68@uabc.edu.mx

____
